# Targeting a therapeutic LIF transgene to muscle via the immune system ameliorates muscular dystrophy

**DOI:** 10.1038/s41467-019-10614-1

**Published:** 2019-06-26

**Authors:** Steven S. Welc, Ivan Flores, Michelle Wehling-Henricks, Julian Ramos, Ying Wang, Carmen Bertoni, James G. Tidball

**Affiliations:** 1 Department of Integrative Biology and Physiology, University of California, Los Angeles, CA 90095-1606 USA; 2 Molecular, Cellular & Integrative Physiology Program, University of California, Los Angeles, CA 90095-1606 USA; 3 Department of Neurology, David Geffen School of Medicine at UCLA, University of California, Los Angeles, CA 90095 USA; 4 Department of Pathology and Laboratory Medicine, David Geffen School of Medicine at UCLA, University of California, Los Angeles, CA 90095 USA

**Keywords:** Inflammation, Skeletal muscle, Diseases, Neuromuscular disease

## Abstract

Many potentially therapeutic molecules have been identified for treating Duchenne muscular dystrophy. However, targeting those molecules only to sites of active pathology is an obstacle to their clinical use. Because dystrophic muscles become extensively inflamed, we tested whether expressing a therapeutic transgene in leukocyte progenitors that invade muscle would provide selective, timely delivery to diseased muscle. We designed a transgene in which leukemia inhibitory factor (LIF) is under control of a leukocyte-specific promoter and transplanted transgenic cells into dystrophic mice. Transplantation diminishes pathology, reduces Th2 cytokines in muscle and biases macrophages away from a CD163+/CD206+ phenotype that promotes fibrosis. Transgenic cells also abrogate TGFβ signaling, reduce fibro/adipogenic progenitor cells and reduce fibrogenesis of muscle cells. These findings indicate that leukocytes expressing a LIF transgene reduce fibrosis by suppressing type 2 immunity and highlight a novel application by which immune cells can be genetically modified as potential therapeutics to treat muscle disease.

## Abstract (web summary)

A number of therapeutic agents aimed at reducing pathology in Duchenne muscular dystrophy have been developed, but may have off-target effects when delivered systemically. Here, the authors express the therapeutic LIF transgene in leukocytes, and show this results in targeting to inflamed dystrophic muscle and reduced fibrosis by suppressing type 2 immunity.

## Introduction

Over recent years, investigators have identified numerous, potentially-therapeutic molecules for the treatment of Duchenne muscular dystrophy (DMD), a lethal and incurable muscle-wasting disease. For example, systemic delivery of therapeutic agents that can inhibit fibrosis (e.g., block TGFβ function^1–3^), inhibit muscle wasting (e.g., myostatin blocking molecules^4^), and increase numbers of muscle stem cells called satellite cells (e.g., Klotho^5^) all reduce pathology in the *mdx* mouse model of DMD. However, systemic delivery of any of these molecules presents risks of unintended off-target effects which provide an obstacle to their clinical application for the treatment of DMD. In addition, the occurrence of muscle pathology is not synchronized in DMD patients. The unpredictable timing and severity of disease vary between muscles in a single individual at any given time, and also vary between locations in a single muscle^6^. Even if a therapeutic agent were specifically targeted to dystrophic muscle, achieving delivery only when pathology is active presents an additional challenge.

Nature has provided a naturally-occurring system for targeted delivery of potentially-therapeutic molecules to dystrophic muscle at stages of the disease when pathology is active. Coinciding with the unpredictable ebb and flow of pathology in muscular dystrophy, inflammatory cells invade in numbers that coincide with the magnitude of muscle pathology. Although the immune cell infiltrate in dystrophin-deficient muscle is complex^7–12^, macrophages comprise the vast majority and they can reach concentrations that exceed 10^7^ cells per pound of muscle at the peak of *mdx* pathology^7^. They are also rich sources of regulatory molecules that can amplify muscle damage but also promote muscle repair and regeneration in muscular dystrophy^7,13,14^. Thus, the introduction of therapeutic transgenes that are expressed at elevated levels in activated macrophages or other immune cells could provide a strategy for intrinsically-regulated targeting of therapeutic molecules specifically to dystrophic muscles at the time of active pathology and at levels that were commensurate with the extent of pathology.

In this investigation, we test whether transplantation of bone marrow cells (BMCs) into which we have introduced a leukemia inhibitory factor (LIF) transgene controlled by the human CD11b promoter reduces the pathology of *mdx* dystrophy. Although *mdx* pathology is less severe than DMD pathology, they share the pathological features of muscle inflammation and progressive fibrosis that persist over the entire lifespan and impair muscle function, reduce health and increase mortality. The CD11b promoter was chosen to drive the therapeutic transgene because CD11b is expressed at low or undetectable levels in myeloid precursors, but at increasingly elevated levels during myeloid cell differentiation and activation^15–17^. LIF was selected as a therapeutic molecule to test this system because it is expressed by macrophages and can influence muscle growth, fibrosis, and inflammation during disease or following injury^18–21^. Our findings show that this intervention significantly modifies intramuscular macrophage phenotype and reduces inflammation and fibrosis of dystrophic muscle, thereby reducing pathology. Perhaps more valuable, the findings indicate that inflammatory cells can be exploited as natural vectors to deliver therapeutic transgenes for the treatment of chronic diseases in which there is a significant inflammatory component.

## Results

### A CD11b regulated LIF transgene suppresses M2-biased markers

**Fig. 1:**
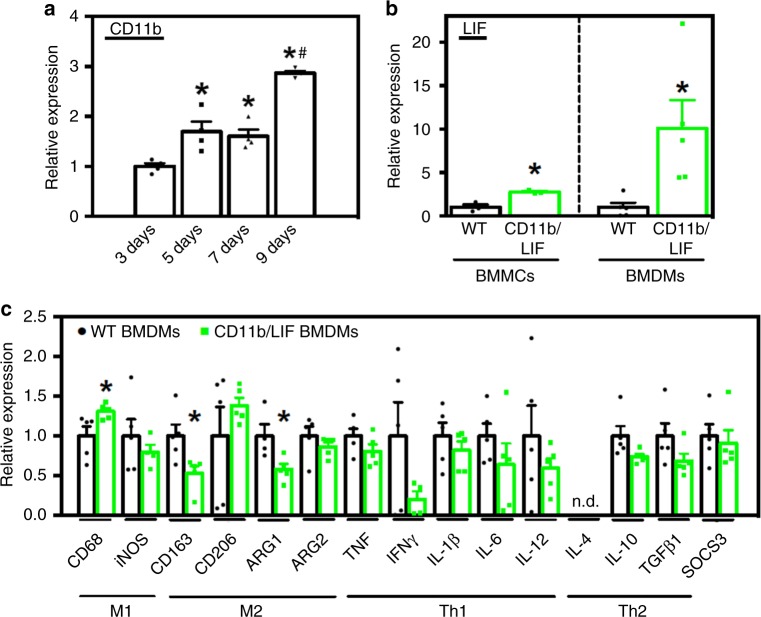
Differentiation of BMCs into macrophages increases CD11b/LIF transgene expression, causing suppression of M2-biased macrophage markers. **a** QPCR data showing differences in the level of *Cd11b* expression in C57BL6 BMCs stimulated with MCSF and differentiated to macrophages for 3–9 days. Values are normalized to 3-day cultures, *n* = 4 for each data set; * indicates significantly different from 3-day data set and # indicates significantly different from 5- and 7-day data sets at *P* < 0.05. *P-*values based on ANOVA with Tukey’s multiple comparison test. For all histograms in the figure, the bars indicate mean ± sem. **b** QPCR data showing increased *Lif* expression in freshly-isolated BMMCs and BMDMs cultured for 9 days from CD11b/LIF transgenic mice compared to transgene negative littermate controls (WT). Data are presented as mean ± sem. BMCs were isolated from three independent donors, *n* = 3 per data set. * Indicates significantly different from WT at *P* < 0.05. *P-*values based on two-tailed *t-*test. *F*-test BMDMs day 9 (*P* = 0.0038). **c** QPCR analysis shows that CD11b/LIF BMDMs have increased the expression of *Cd68* and reduced the expression of *Cd163* and *Arg1*. Data are presented as mean ± sem, *n* = 5 for each data set, *n* = 4 for WT BMDMs *Inos*, and CD11b/LIF BMDMs *Arg1* data sets (*P* < 0.05). n.d. indicates that no expression was detected. Data presented for BMDMs (**b**, **c**) were isolated from a single donor animal of each genotype and cultured as *n* = 5 technical replicates. Significant findings were verified with biological replicates of experiments from independent donors. * Indicates significantly different from WT BMDMs at *P* < 0.05. *P-*values based on two-tailed *t-*test. *F*-test *Cd206* (*P* = 0.0258) and *Il10* (*P* = 0.0311). Source data are provided as a Source Data file

We generated mice with a LIF transgene under control of the CD11b promoter (CD11b/LIF transgenic mice). Quantitative PCR (QPCR) analysis of *Cd11b* mRNA levels confirmed that *Cd11b* expression increased as BMCs differentiate into bone marrow-derived macrophages (BMDMs) (Fig. 1a). Freshly-isolated bone marrow mononuclear cells (BMMCs) from transgenic *mice* had a ~2.8-fold higher *Lif* expression compared to wild-type (WT). After 9 days of culture, *Lif* expression was ~10-fold higher in transgenic BMDMs than WT (Fig. 1b). Thus, LIF transgene expression increased with increased CD11b promoter activation as monocytes differentiate into mature macrophages. Upon becoming fully-differentiated macrophages, the CD11b/LIF transgene had an autocrine effect on macrophage phenotype, increasing expression of *Cd68* by ~31% and reducing *Cd163* and arginase-1 (*Arg1*) by 47% and 42%, respectively (Fig. 1c). CD68 is present at high levels in macrophages that are biased to a pro-inflammatory phenotype (M1-biased). Arginase and CD163 are present in macrophages that are biased toward a pro-fibrotic and reparative phenotype (M2-biased)^22^.

### CD11b/LIF transgene reduces mdx muscle inflammation and fibrosis

**Fig. 2:**
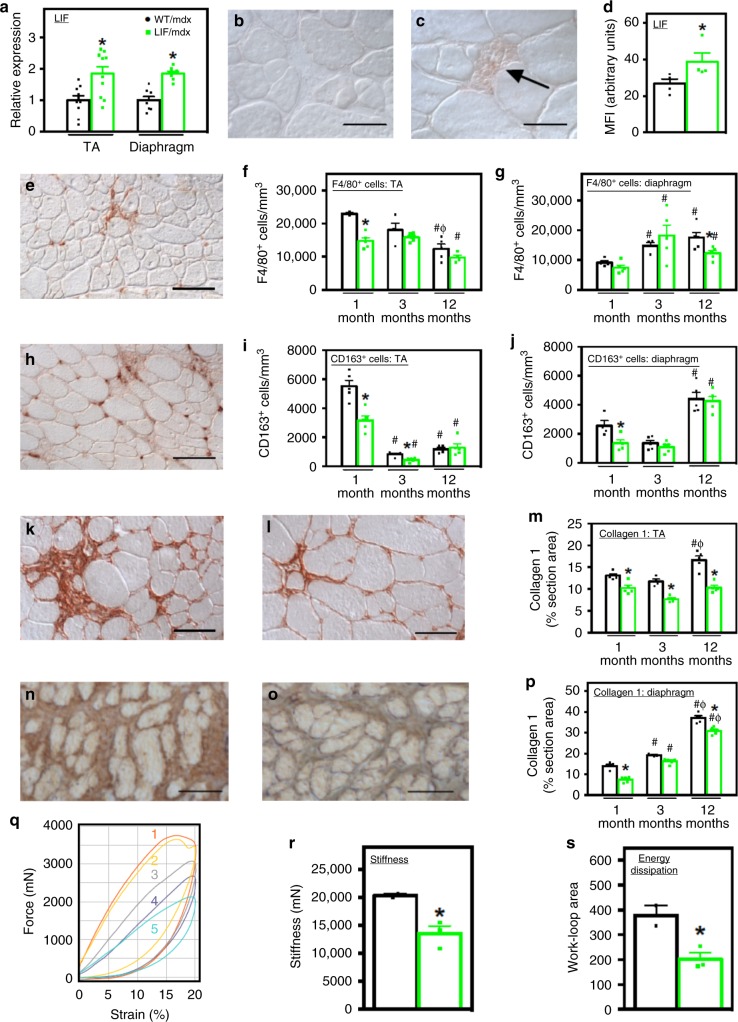
CD11b/LIF transgene expression modulates inflammation and reduces fibrosis. **a** QPCR data showing *Lif* expression in muscles of CD11b/LIF transgenic *mdx* mice (LIF/*mdx*) and non-transgenic littermates (WT/*mdx*), normalized to WT/*mdx*. TA muscles: *n* = 10. Diaphragm muscles: *n* = 8 or 7 for WT/*mdx* and LIF/*mdx* data sets, respectively. * Indicates significant difference versus WT/*mdx* (*P* < 0.05). For all histograms, bars indicate mean ± sem. **b**, **c** Cross-sections of WT/*mdx* (**b**) and LIF/*mdx* (**c**) TA muscles labeled with anti-LIF. Bars = 50 μm. **d** Mean fluorescence intensity (MFI) of inflammatory lesions in sections immunolabeled for LIF. * Indicates significant difference from WT/*mdx* (*n* = 4; *P* < 0.05). **e**–**j** Cross-sections of muscles from WT/*mdx* and LIF/*mdx* mice were immunolabeled with antibodies to F4/80 (**e**) and CD163 (**h**). Numbers of F4/80+ (**f**, **g**) and CD163+ (**i**, **j**) cells were normalized to muscle volume. Labeling of F4/80+ (**e**) and CD163+ (**h**) cells in TA muscle from 1-month-old WT/*mdx* muscle. Bars = 100 μm. *N* = 5 for each group, except *n* = 4 for F4/80 WT/*mdx* 1- and 12-month TA, LIF/*mdx* 12-month TA, WT/*mdx* 1- and 3-month diaphragm, and CD163 WT/*mdx* 12-month TA data sets. **k**–**p** Cross-sections of TA (**k**, **i**) and diaphragm (**n**, **o**) muscles from 12-month-old WT/*mdx* (**k**, **n**) and LIF/*mdx* (**l**, **o**) mice were immunolabeled with anti-collagen type 1. Bars = 50 μm. The volume fraction of muscle occupied by collagen type 1 (**m**, **p**). *N* = 5 for each group, except *n* = 4 for 3-month TA. * Indicates significant difference versus age-matched WT/*mdx* mice (*P* < 0.05). # and Φ indicate significant difference versus 1- and 3-months-old, genotype-matched mice, respectively (*P* < 0.05). *P*-values based on two-tailed *t*-test. **q**–**s** The passive mechanical properties of TA muscles of WT/*mdx* (curves 1 and 2) and LIF/*mdx* (curves 3–5) mice were measured in-situ. Lissajous curves (**q**) show passive stiffness (**r**) and energy dissipation (**s**) of TAs. *N* = 2 and 3 for WT/*mdx* and LIF/*mdx* groups, respectively. * Indicates significant difference versus WT/*mdx* mice. *P*-values based on two-tailed *t*-test. Source data are provided as a Source Data file

We assayed whether the expression of the CD11b/LIF transgene affected *mdx* pathology, focusing on influences on muscle inflammation and fibrosis. We confirmed elevated expression of *Lif* in the tibialis anterior (TA) and diaphragm muscles of transgenic mice (CD11b/LIF *mdx* mice) (Fig. 2a) and observed that cells in inflammatory lesions in CD11b/LIF *mdx* mice showed higher levels of LIF protein than non-transgenic mice (Fig. 2b–d). However, sera from transgenic mice showed no elevation in LIF protein assayed by ELISA (mean ± sem: WT/*mdx* 19.25 ± 1.85 and LIF/*mdx* 26.19 ± 4.86 pg/ml , *n* = 3 per data set, *P* = 0.25; two-tailed *t*-test). We also found no significant differences in the concentrations of cytokines previously implicated in influencing the pathology of muscular dystrophy (IFNγ, TNF, IL-4, and IL-10) in the serum of transgenic mice, compared to non-transgenic mice (Supplementary Fig. 1).

We assessed effects of the transgene on *mdx* pathology over the course of the disease, sampling at the acute onset of pathology (1-month-old), the period of successful regeneration (3-months-old), and the late, progressive stage of pathology (12-months-old) in TA muscles. Diaphragm muscles show a progressive pathology following disease onset. The CD11b/LIF transgene reduced numbers of macrophages expressing the pan-macrophage marker F4/80 at the stages of pathology characterized by extensive, muscle inflammation (1-month-old in TA; 12-months-old in diaphragm) (Fig. 2e–g). The transgene also reduced numbers of CD163+ macrophages at the acute onset of pathology in both TA and diaphragm (Fig. 2h–j) but did not affect numbers of CD68+ macrophages in either muscle at any stage of the disease that we tested (Supplementary Fig. 2).

We tested whether the CD11b/LIF transgene reduced collagen accumulation in *mdx* muscles, which would be consistent with a reduction in numbers or activity of CD163 macrophages that promote fibrosis of dystrophic muscle^23^. Both the TA and diaphragm showed significant reductions in collagen type 1 at the acute onset of pathology, and the transgene completely abrogated collagen type 1 accumulation in the TA muscle, at least until 12-months-old (Fig. 2k–m). The CD11b/LIF transgene also reduced accumulation of collagen type 1 in diaphragms (Fig. 2n–p) and reduced accumulation of collagen types 3 and 5 in diaphragms at late stages of pathology and reduced collagen type 5 in 3-month-old TA muscles (Supplementary Fig. 3).

Because the CD11b/LIF transgene prevented collagen type 1 accumulation in TA muscles and collagen type 1 is primarily responsible for increased muscle stiffness caused by fibrosis, we assayed for changes in the passive mechanical properties of TA muscles in CD11b/LIF transgenic *mdx* mice. We subjected TA muscles to cyclic, dynamic loading using 20% strains at a 0.6/s strain rate, which is within the physiological range. Lissajous figures obtained by measuring force–strain relationships showed that muscle stiffness (indicated by the slope of the tangent to the loading phase of each cycle) was significantly less in CD11b/LIF transgenic *mdx* mice (Fig. 2q, r). In addition, the transgenic *mdx* muscles showed less energy dissipation during each cycle of loading (proportional to the area inside each hysteresis loop during a cycle of loading/unloading) (Fig. 2q, s), indicating higher mechanical efficiency in the CD11b/LIF transgenic muscles.

### CD11b/LIF transgene does not impair muscle growth

Although previous investigations showed that M2-biased macrophages promote muscle fibrosis, they also promote regeneration^23,24^. We tested this possibility by assaying for effects of the transgene on TA muscle fiber cross-sectional area as an index of regeneration and found no difference in TA fiber size between transgenic and non-transgenic *mdx* mice at any age sampled (Supplementary Fig. 4A). We also assayed for the proportion of muscle fibers that expressed developmental myosin heavy chain (dMHC), which is upregulated in regenerating fibers. We observed a higher proportion of dMHC+ fibers in TAs of CD11b/LIF transgenic *mdx* mice at 3-months-old and a trend for more dMHC+ fibers at 1-month and 12-months-old, compared to non-transgenic *mdx* mice (Supplementary Fig. 4B). Similarly, the proportions of dMHC+ fibers in 3-months-old and 12-months-old diaphragms were increased by the transgene (Supplementary Fig. 4C). Collectively, these observations indicate that the CD11b/LIF transgene does not impair muscle growth or regeneration, despite the reduction of CD163+ cells.

### Transplanted CD11b/LIF cells reduce inflammation

Our analyses of CD11b/LIF transgenic *mdx* mice showed that the transgene reduces muscle inflammation and fibrosis, thereby validating the transgene as a therapeutic molecule for muscular dystrophy. However, our primary goal in the investigation was to determine whether transplanted bone marrow derived cells (BMDCs) could serve as vehicles to deliver therapeutic molecules to dystrophic muscle through a clinically-relevant approach; in particular, we questioned whether transplantation of genetically-modified BMCs into dystrophic animals provides a strategy for targeted delivery of therapeutic cargo to diseased muscle. We assayed treatment effects in 6-months-old *mdx* mice at 4 months post-bone marrow transplantation (BMT) for scientific and technical reasons. First, we anticipated that a likely, beneficial outcome of leukocyte delivery of a LIF transgene to muscle would be reductions in fibrosis. Our previous work^5^ showed that 6-months-old *mdx* muscles show significantly elevated accumulation of type I and type III collagen. We also showed that at 6-months-old, *mdx* limb muscles contain elevated numbers of M2-biased macrophages that contribute to muscle fibrosis^5^. The technical rationale for sampling at 6 months is that engraftment of transplanted cells takes time and our preliminary experiments showed that high levels of engraftment could be achieved by 4 months post-BMT.

**Fig. 3:**
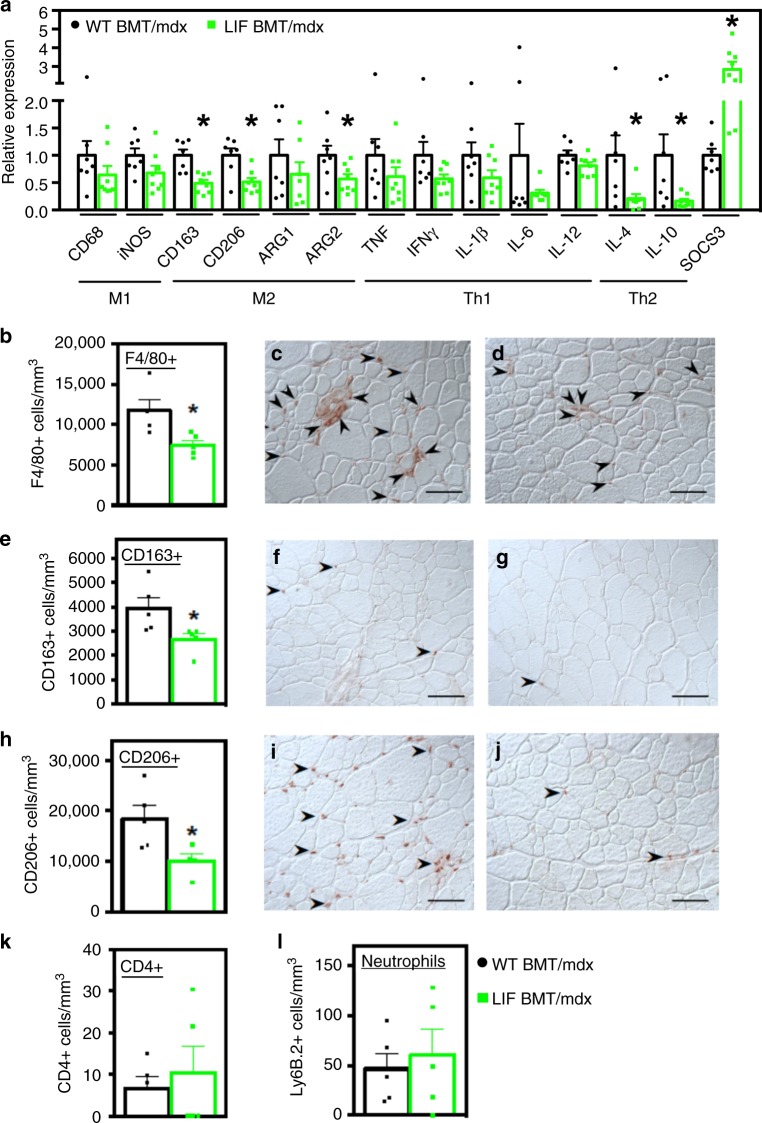
Transplantation of CD11b/LIF transgenic BMCs into *mdx* mice reduces inflammation in dystrophic muscle. **a** QPCR analysis shows that the transplantation of CD11b/LIF transgenic BMCs into *mdx* recipients (LIF BMT/*mdx*) reduced expression of transcripts associated with M2-biased macrophages (*Cd163*, *Cd206*, and *Arg2*), Th2 cytokines (*Il4* and *Il10*), and increased expression of the negative regulator of cytokine signaling (*Socs3)* compared to WT BMT *mdx* recipients (WT BMT/*mdx*) 4 months post-transplantation. *N* = 7 or 8 for WT BMT/*mdx* and LIF BMT/*mdx* data sets, respectively, except *n* = 7 for LIF BMT/*mdx Arg1* data set. * Indicates significantly different from WT BMT/*mdx* recipients at *P* < 0.05. *F*-test *Ifng* (*P* = 0.0145), *Il6* (*P* < 0.0001), *Il4* (*P* = 0.0015), *Il10* (*P* < 0.0001), and *Socs3* (*P* = 0.0061). For all histograms in the figure, the bars indicate mean ± sem. **b**–**j** Cross-sections of TA muscles from WT BMT/*mdx* (**c**, **f**, **i**) or LIF BMT/*mdx* (**d**, **g**, **j**) mice were immunolabeled with antibodies to F4/80 (**c**, **d**), CD163 (**f**, **g**), and CD206 (**i**, **j**). Bars = 50 μm. The numbers of F4/80+ (**b**), CD163+ (**e**), and CD206+ (**h**) cells normalized to muscle volume were reduced in LIF BMT/*mdx* recipients. Similarly, cross-sections were immunolabeled with antibodies to CD4 and Ly-6B.2 (neutrophils) to test for changes in the concentrations of other populations of immune cells. There was no change in the concentrations of CD4+ (**k**) and Ly-6B.2+ (**l**) cells. *N* = 5 for each data set, except *n* = 4 for CD206 LIF BMT/*mdx* data set. * Indicates significantly different from WT BMT/*mdx* recipients at *P* < 0.05. All *P-*values based on two-tailed *t-*test. Source data are provided as a Source Data file

At the time of tissue collection from transplant recipients, circulating leukocytes were 86.6% donor-derived (sem = 1.14; *n* = 25). QPCR of muscles showed that CD11b/LIF recipients (LIF BMT/*mdx* mice) had reduced expression of the M2-biased markers *Cd163*, CD206 (*Mrc1*), and arginase-2 (*Arg2*) expression by 51%, 49%, and 43%, respectively (Fig. 3a). This effect resembles the autocrine effect of CD11b/LIF on macrophages in vitro (Fig. 1c). Additionally, the transgene affected the expression of Th2 cytokines associated with M2-biased macrophage activation, IL-4 (*Il4*) and IL-10 (*Il10*), which were reduced by ~79% and ~84%, respectively (Fig. 3a). Reduced cytokine expression was accompanied by a ~2.8-fold increase in the expression of suppressor of cytokine signaling 3 (*Socs3*) in CD11b/LIF BMT recipients (Fig. 3a). *Socs3* expression is activated by LIF^25^ and its elevation in muscles of CD11b/LIF BMT recipients verifies an increase in LIF signaling in muscle.

We tested the effect of CD11b/LIF BMT on macrophage numbers and phenotype because changes in macrophages have profound effects on dystrophic muscle pathology^7,13,14,26^. We performed immunohistochemistry using anti-F4/80, to identify total macrophage populations, or anti-CD68 (M1-biased macrophages), anti-CD163 (M2-biased), or anti-CD206 (M2-biased). *Mdx* mice that received CD11b/LIF BMCs had 37% fewer F4/80+ cells compared to mice receiving WT BMCs (Fig. 3b). Quantitation of CD68+, CD163+ and CD206+ macrophages showed no difference in CD68+ cells (mean ± sem: WT BMT/*mdx* 17,525 ± 1502 and LIF BMT/*mdx* 16,377 ± 1440 cells/mm^3^, *n* = 5 per data set, *P* = 0.60; two-tailed *t*-test), a 32% reduction of CD163+ cells (Fig. 3e) and 46% fewer CD206+ cells (Fig. 3h) in the dystrophic muscle. However, numbers of CD4+ T-cells and neutrophils in *mdx* muscles were unaffected by transplantation of CD11b/LIF BMCs (Fig. 3k, l), indicating a selective reduction of M2-biased macrophages caused by transgenic BMDCs.

### LIF reduces *Ccl2* expression in muscle and macrophages

**Fig. 4:**
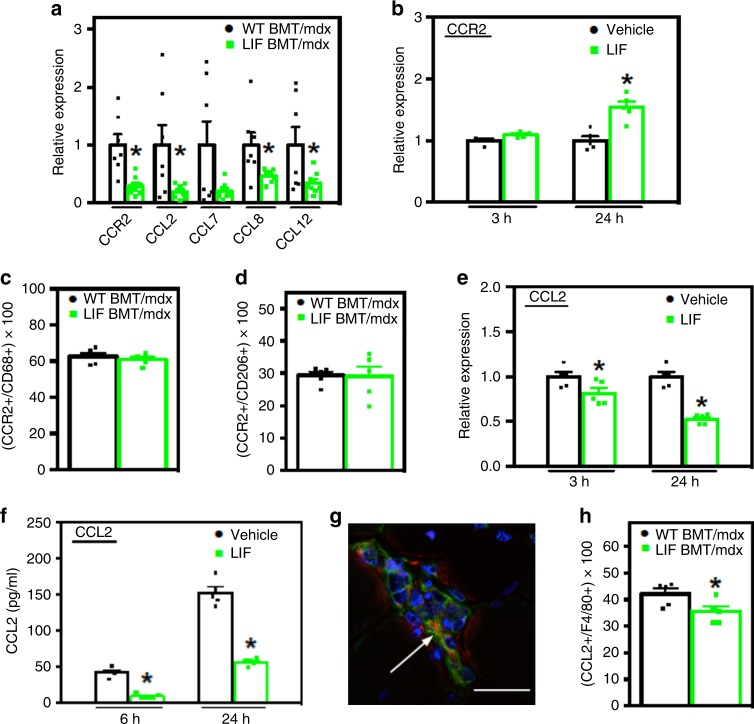
Transplantation of CD11b/LIF transgenic BMCs disrupts *Ccl2* expression in dystrophic muscles by inhibiting macrophage expression of CCL2. **a** QPCR analysis shows that TA muscles from LIF BMT/*mdx* recipients have reduced expression of *Ccr2* and its ligands *Ccl2*, *Ccl7* (*P* = 0.06), *Ccl8*, and *Ccl12*. *N* = 7 or 8 for WT BMT/*mdx* and LIF BMT/*mdx* data sets, respectively, except *n* = 7 for LIF BMT/*mdx Ccl8* data set. * Indicates significantly different from WT BMT/*mdx* recipients at *P* < 0.05. *F*-test *Ccr2* (*P* = 0.0087), *Ccl2* (*P* *<* 0.0001), *Ccl7* (*P* = 0.0001), *Ccl8* (*P* = 0.001), and *Ccl12* (*P* = 0.001). For all histograms in the figure, the bars indicate mean ± sem. **b** QPCR analysis for *Ccr2* gene expression of BMDMs treated with recombinant LIF (10 ng/ml) for 3- and 24-h. **c**, **d** Muscle sections co-labeled with antibodies to CD68 (**c**) or CD206 (**d**) and CCR2 show no change in the proportions of cells co-expressing CCR2 between transplant recipient groups. *N* = 5 for each data set. **e** QPCR analysis shows reduced *Ccl2* gene expression in BMDMs stimulated with LIF as described in (**b**). **f** ELISA of conditioned media showed less CCL2 secreted into the media of BMDMs stimulated with LIF for 6- and 24-h compared to control cultures. For cell culture experiments, *N* = 5 technical replicates for each data set, cells for each time point were isolated from independent donors. Significant findings were verified with biological replicates of experiments from independent donors. *P-*values based on two-tailed *t-*test. *F*-test CCL2 protein 24 h (*P* = 0.0337). **g**, **h** Cross-sections of TA muscles from WT BMT/*mdx* or LIF BMT/*mdx* mice were immunolabeled with antibodies to F4/80 (green) and CCL2 (red) show that F4/80+ cells express CCL2 (**g**). Nuclei are stained blue with DAPI. Bar = 10 μm. **h** The proportion of F4/80+ cells co-expressing CCL2 was reduced in LIF BMT/*mdx* recipients. *N* = 5 for each data set, * indicates significantly different from WT BMT/*mdx* recipients at *P* *<* 0.05. *P-*values based on two-tailed *t-*test. Source data are provided as a Source Data file

The large reductions of M2-biased macrophages in dystrophic muscle of mice transplanted with CD11b/LIF BMCs (Fig. 3) suggest that LIF inhibits their recruitment. Because abrogation of CCR2 signaling reduces macrophage accumulation in dystrophic muscle^27^, we tested whether CCR2 signaling was affected by LIF. QPCR assays showed reduced expression of Ccr2 and its ligands *Ccl2*, *Ccl8* and *Ccl12* in muscles of CD11b/LIF BMT recipients, and a strong trend for lower levels of *Ccl7* expression (*P* = 0.06) (Fig. 4a).

We next tested the possibility that LIF acts directly on macrophages to inhibit CCR2 signaling in vitro. Unexpectedly, brief periods of macrophage stimulation with LIF had no effect on *Ccr2* expression and extended periods significantly increased *Ccr2* expression (Fig. 4b). We assayed whether the CD11b/LIF transgene affected the numbers of intramuscular macrophages that expressed detectible CCR2 but found that the proportion of CD68+ or CD206+ macrophages that expressed CCR2 was not influenced by the transgene (Fig. 4c, d). This indicates that reductions in macrophage-derived CCR2 in muscles reflect reductions in macrophage numbers, rather than ablating the expression of CCR2 in macrophages in CD11b/LIF BMT recipients. However, stimulation of BMDMs with LIF reduced *Ccl2* expression and CCL2 protein secretion (Fig. 4e, f), indicating that LIF acts directly on macrophages to negatively regulate *Ccl2*. In addition, F4/80+ macrophages were prominent sources of CCL2 in *mdx* muscle (Fig. 4g), and transplantation of CD11b/LIF BMCs reduced the proportion of F4/80+ macrophages that expressed detectible CCL2 by 15% (Fig. 4h).

### Transplanted CD11b/LIF cells reduce muscle fibrosis

**Fig. 5:**
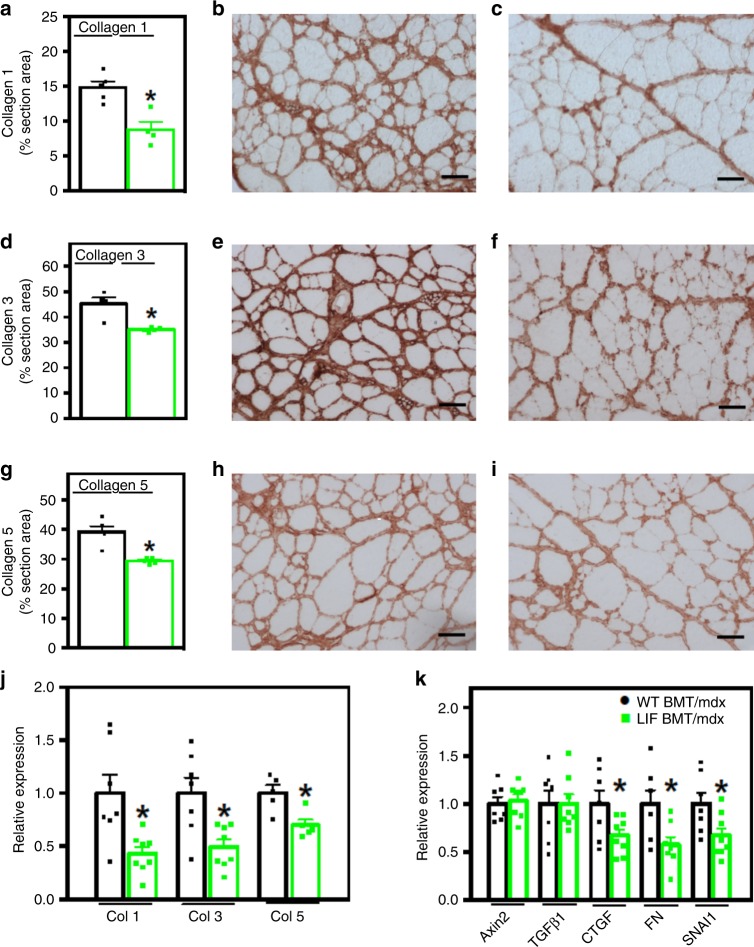
Transplantation of CD11b/LIF transgenic BMCs into *mdx* mice reduces muscle fibrosis. **a**–**i** TA muscles from WT BMT/*mdx* (**b**, **e**, **h**) and LIF BMT/*mdx* transplant recipients (**c**, **f**, **i**) were immunolabeled for collagen types 1 (**a**–**c**), 3 (**d**–**f**), and 5 (**g**–**i**). Bars = 50 µm. The volume fraction of muscle occupied by collagen types 1 (**a**), 3 (**d**), and 5 (**g**) was reduced in LIF BMT/*mdx* recipients. *N* = 5 for WT BMT/*mdx* and LIF BMT/*mdx* data sets, except *n* = 4 WT BMT/*mdx* collagen type 3 and LIF BMT/*mdx* collagen type 1. * Indicates significantly different from WT BMT/*mdx* recipients at *P* < 0.05. *F*-test collagen type 3 (*P* = 0.0055) and type 5 (*P* = 0.0155). For all histograms in the figure, the bars indicate mean ± sem. **j** QPCR data presented as mean ± sem shows that LIF BMT/*mdx* recipients also had reduced expression of transcripts encoding *Col1a1*, *Col3a1*, and *Col5a3*. *N* = 7 or 8 for WT BMT/*mdx* and LIF BMT/*mdx* data sets, respectively, except *n* = 5 for *Col5a3* data sets. **k** QPCR analysis of transcripts associated with the pro-fibrotic Wnt- (*Axin2*) and TGFβ1-signaling (*Tgfb1*, *Ctgf*, *Fn1*, and *Snai1*) pathways showed reduced expression of *Ctgf*, *Fn1*, and *Snai1* in LIF BMT/*mdx* recipients. *N* = 7 or 8 for WT BMT/*mdx* and LIF BMT/*mdx* data sets, respectively, except *n* = 7 for LIF BMT/*mdx Axin2* group. * Indicates significantly different from WT BMT/*mdx* recipients at *P* < 0.05. *P-*values based on two-tailed *t-*test. *F*-test *Col1a1* (*P* = 0.0250). Source data are provided as a Source Data file

Fibrosis of dystrophin-deficient muscle is largely driven by arginine metabolism by arginase expressed by M2-biased macrophages^23^. Arginine hydrolysis by arginase produces metabolites that are utilized to generate substrate molecules necessary for connective tissue production^28^. Because we observed reductions in M2-biased macrophages in muscles of *mdx* mice that were recipients of CD11b/LIF BMT and lower levels of expression of *Arg2*, we assayed whether fibrosis was affected. Transplantation of CD11b/LIF BMCs reduced collagen types 1, 3, and 5 in *mdx* muscle by ~41%, 22%, and 25%, respectively, compared to WT BMT recipients (Fig. 5a–i). However, the anti-fibrotic effect of CD11b/LIF BMT cannot be solely attributed to reductions of arginine metabolism by M2-biased macrophages. QPCR data showed that mRNA levels of collagen types 1 alpha 1 (*Col1a1*), 3 alpha 1 (*Col3a1*), and 5 alpha 3 (*Col5a3*) were reduced by ~57%, 51%, and 30%, respectively, in CD11b/LIF BMC recipients (Fig. 5j), indicating treatment effects on fibrogenic cells, in addition to effects on macrophages that provide substrate for fibrogenesis.

M2-biased macrophages can act directly on fibrogenic cells through TGFβ which activates fibro/adipogenic progenitor cells (FAPs) into fibroblasts and stimulates fibroblasts to produce collagen^29–32^. TGFβ can also activate Wnt-signaling, which increases myogenic-to-fibrogenic conversion of muscle stem cells, further contributing to dystrophic muscle fibrosis^33^. We tested whether the CD11b/LIF BMT affected key transcripts of the Wnt and TGFβ pro-fibrotic pathways. Although there was no effect on the expression of *Tgfb1* or *Axin2*, a Wnt-target gene (Fig. 5k), the expression of downstream TGFβ target genes connective tissue growth factor (*Ctgf*), fibronectin (*Fn1*), and snail family zinc finger 1 (*Snai1*)^34–37^ were reduced by ~33%, 43%, and 33%, respectively (Fig. 5k).

### LIF reduces macrophage TGFβ1 expression

**Fig. 6:**
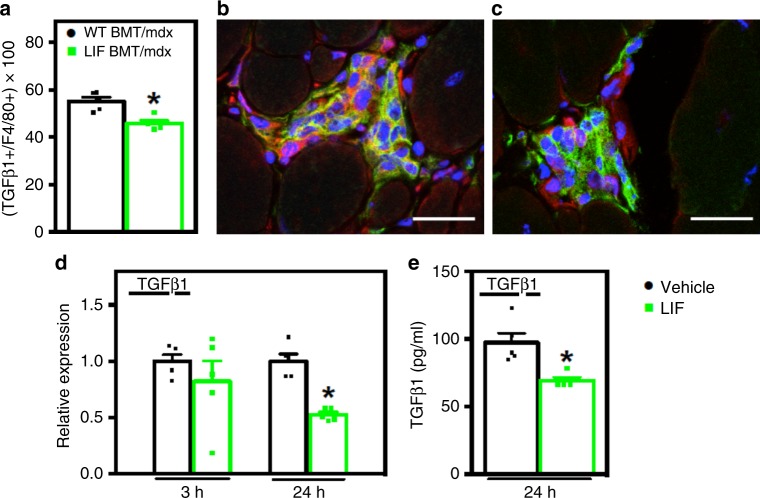
LIF inhibits macrophage TGFβ1 expression. **a**–**c** Muscle sections were co-labeled with antibodies to pro-fibrotic TGFβ (red) and the pan macrophage marker F4/80 (green) to test for changes in macrophage expression of TGFβ. Nuclei appear blue (DAPI). Bars = 25 μm. **a** The proportion of F4/80+ cells co-expressing TGFβ was reduced in LIF BMT/*mdx* recipients. The greatest reduction in the number of F4/80+ cells positive for TGFβ (orange) was in inflammatory lesions of LIF BMT/*mdx* (**c**) compared to WT BMT/*mdx* recipients (**b**). *N* = 5 for each data set, * indicates significantly different from WT BMT/*mdx* recipients at *P* < 0.05. For all histograms in the figure, the bars indicate mean ± sem. **d** QPCR analysis of BMDMs treated with recombinant LIF (10 ng/ml) for 3- or 24-h shows that *Tgfb1* expression is inhibited by LIF after 24 h of stimulation. **e** The concentration of secreted TGFβ was also reduced in BMDMs stimulated with LIF for 24 h, analyzed by ELISA. *N* = 5 technical replicates for each data set. Significant findings were verified with biological replicates of experiments from independent donors. * Indicates significantly different from control at *P* < 0.05. Source data are provided as a Source Data file

Although we observed no effect of CD11b/LIF BMT on *Tgfb1* mRNA in whole muscle homogenates, we assayed more specifically for effects on TGFβ expression in intramuscular macrophages by assaying the proportion of macrophages that expressed TGFβ. We found that there were 17.7% fewer intramuscular macrophages that expressed detectible TGFβ in CD11b/LIF recipients, compared to WT recipients (Fig. 6a). Interestingly, the greatest reduction of TGFβ expressing macrophages was seen in inflammatory lesions of CD11b/LIF recipients (Fig. 6c) compared to WT recipients (Fig. 6b).

We tested whether reduced TGFβ1 expression in CD11b/LIF BMT recipients reflected direct actions of LIF on macrophages to inhibit TGFβ1 expression. When we treated BMDMs with LIF for 24 h, *Tgfb1* gene expression was reduced by 47% and secreted TGFβ protein expression by 29% (Fig. 6d, e), showing that LIF is a negative regulator of TGFβ1 expression in macrophages. However, *Tgfb1* gene expression was not reduced after 3 h of LIF stimulation, suggesting that LIF-mediated inhibition of *Tgfb1* could be a secondary effect.

### LIF reduces fibrogenesis and *Ctgf* mRNA in muscle cells

**Fig. 7:**
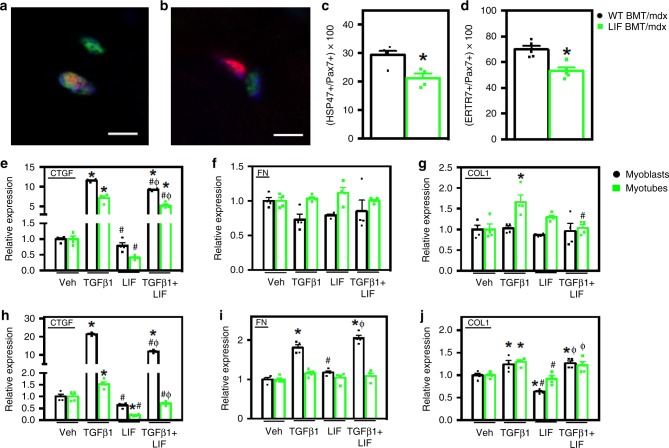
LIF inhibits fibrogenesis and TGFβ1-induced *Ctgf* expression in muscle cells. **a** TA muscle sections were co-labeled with antibodies to Pax7 (red) and HSP47 (green) in WT BMT/*mdx* (**a**) and LIF BMT/*mdx* (**b**) recipients. Nuclei appear blue (DAPI). Bars = 5 μm. **c** Fewer Pax7+ cells co-expressed HSP47 in LIF BMT/*mdx* recipients (green symbols) compared to WT BMT/*mdx* recipients (black symbols). **d** Muscle sections were also co-labeled with antibodies to Pax7 and fibrogenic marker Ertr7 to confirm that fewer Pax7+ cell acquired a fibrogenic phenotype in LIF BMT/*mdx* recipients. *N* = 5 for each data set, except *n* = 4 for WT BMT/*mdx* Pax7/HSP47 data set, * indicates significantly different from WT BMT/*mdx* at *P* < 0.05. *P-*values based on two-tailed *t-*test. For all histograms in the figure, the bars indicate mean ± sem. **e**–**j** Myoblasts (black symbols) and myotubes (green symbols) were stimulated with LIF (10 ng/ml) and TGFβ1 (10 ng/ml) for 3- (**e**–**g**) or 24-h (**h**–**j**). **e**, **h** LIF inhibited TGFβ1-induced *Ctgf* mRNA in myoblasts and myotubes after 3- and 24-h of stimulation. LIF inhibited basal *Ctgf* expression in myotubes at 24 h (**h**). **f**, **i** LIF did not affect *Fn1* expression in myoblasts or myotubes after 3- or 24-h. Additionally, LIF attenuated TGFβ1-induced *Col1a1* expression in myotubes, but not myoblasts after 3 h of stimulation (**g**). Myoblasts stimulated with LIF for 24 h had reduced *Col1a1* expression (**j**). *N* = 4 technical replicates per group. Significant findings were verified with biological replicates of experiments from independent cultures. * Indicates significantly different from control, # indicates significantly different from TGFβ1-stimulated, and Φ indicates significantly different from LIF-stimulated at *P* < 0.05. *P-*values based on ANOVA with Tukey’s multiple comparison test. Source data are provided as a Source Data file

TGFβ signaling promotes the fibrogenic conversion of myogenic cells in dystrophic muscle, thereby contributing to fibrosis^33^. Because transplantation of CD11b/LIF BMCs into *mdx* mice reduces fibrosis, we tested whether LIF reduces the proportion of myogenic cells acquiring a fibrogenic phenotype. Muscle sections that were double-labeled with anti-Pax7, a marker of satellite cells, and anti-HSP47, a collagen-specific molecular chaperone^38,39^ showed that the proportion of Pax7+ cells that expressed HSP47 was reduced by 27.8% in CD11b/LIF recipients (Fig. 7a–c); this indicates that satellite cells had a less fibrogenic phenotype in CD11b/LIF recipients. Expression of *Serpinh1*, the gene that encodes HSP47, was also reduced 24% in the whole muscle lysate of CD11b/LIF recipients (mean ± sem: WT BMT/*mdx* 1 ± 0.08 and LIF BMT/*mdx* 0.76 ± 0.06, *n* = 7 and 8 per data set, respectively, *P* = 0.03; two-tailed *t*-test*)*. We also assayed whether transplantation of CD11b/LIF BMCs affected the proportion of satellite cells that expressed ERTR7 in vivo. ERTR7 was chosen in addition to HSP47 because satellite cells in injured and aging muscle that display elevated levels of ERTR7 expression have shifted away from a myogenic phenotype, toward a fibrogenic phenotype^40,41^. Our data show that the transgene reduced the proportion of satellite cells that expressed ERTR7 in *mdx* muscle in vivo, similar to the reduction of satellite cells expressing HSP47 (Fig. 7c, d).

We also tested whether the CD11b/LIF transgene affected the phenotype of myogenic progenitor cells (MPCs) in later stages of *mdx* pathology by assaying for changes in the expression of fibrogenic genes in MPCs that were freshly-isolated from muscles of 14-months-old mice. MPCs (CD11b-CD31-CD45-Sca1-α7 integrin+ cells) from CD11b/LIF transgenic *mdx* mice showed lower expression levels of *Fn1* and *Col3a1* compared to non-transgenic mice (Supplementary Fig. 5). In addition, we observed strong trends for the reduction in expression of *Serpinh1* (HSP47) and *Col1a1* in freshly-isolated MPCs.

We also examined the effects of LIF on TGFβ1-induced muscle cell fibrogenesis in vitro. We assayed myoblasts and myotubes treated with TGFβ1 and/or LIF for changes in expression of fibrogenic genes downregulated in CD11b/LIF BMT recipients (*Ctgf*, *Fn1*, and *Col1a1*; Fig. 5j, k). Co-stimulation with TGFβ1 and LIF inhibited *Ctgf* expression, compared to cells treated with TGFβ1 only (Fig. 7e, h). LIF also reduced basal *Ctgf* expression after 24 h of stimulation in myotubes. *Fn1* expression was stable in myoblasts treated with TGFβ1, LIF, or TGFβ1 + LIF for 3 h (Fig. 7f). After 24 h, TGFβ1-induced *Fn1* expression, but co-stimulation with LIF had no effect (Fig. 7i). TGFβ1 stimulation for 3 h induced the expression of *Col1a1* in myotubes, and LIF attenuated TGFβ1-induced expression *of Col1a1* in myotubes (Fig. 7g). LIF stimulation for 24 h reduced basal *Col1a1* expression in myoblasts, but not TGFβ1-induced expression of *Col1a1* (Fig. 7j).

### LIF reduces the prevalence of FAPs in dystrophic muscle

**Fig. 8:**
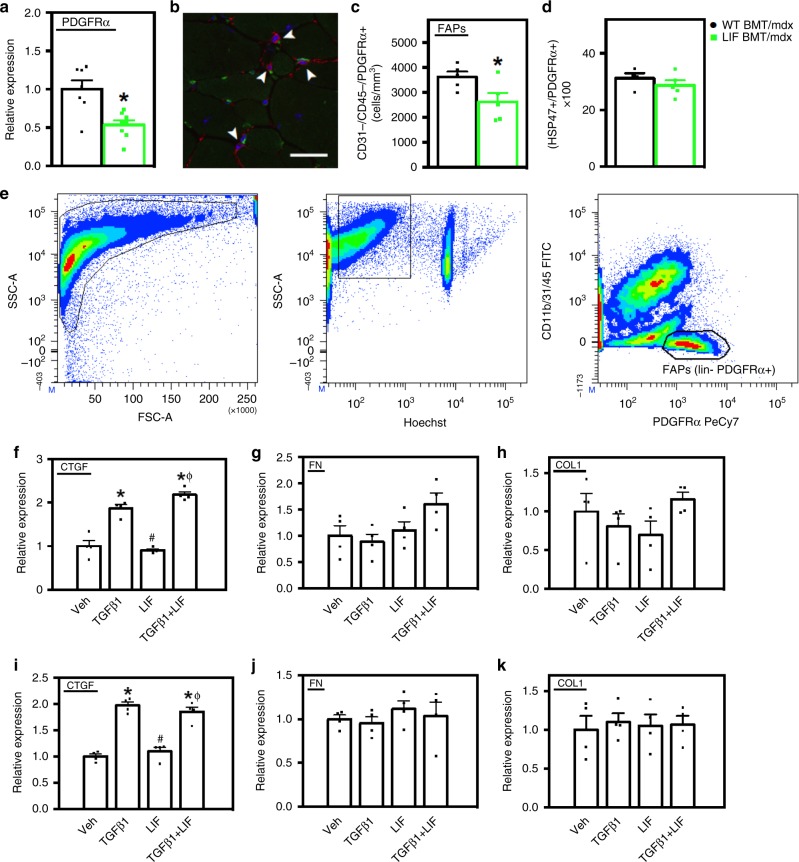
Transplantation of CD11b/LIF transgenic BMCs reduces the numbers of FAPs in dystrophic muscle but does not affect phenotype. **a** QPCR analysis shows that TA muscles from LIF BMT/*mdx* recipients have reduced *Pdgfra* gene expression. *N* = 7 or 8 for WT BMT/*mdx* and LIF BMT/*mdx* data sets, respectively, * indicates significantly different from WT BMT/*mdx* recipients at *P* < 0.05. *P-*values based on two-tailed *t-*test. For all histograms in the figure, the bars indicate mean ± sem. **b** To quantify the number of FAPs, muscle sections were co-labeled with antibodies to PDGFRα (red) and CD31, CD45 (green). Arrowheads indicate FAPs (CD31-CD45-PDGFRα+). Bar = 50 μm. **c** Fewer FAPs (CD31-CD45-PDGFRα+) in TA cross-sections of LIF BMT/*mdx* recipients compared to WT BMT/*mdx* recipients. *N* = 5 for each data set. **d** There was no detectible change in phenotype of PDGFRα+ cells assayed for co-expression of the fibrogenic marker HSP47. **e** FACS plots demonstrating strategy for sorting FAPs (Hoechst + CD11b-CD31-CD45-PDGFRα+). Fibroblasts derived from FAPs were stimulated with LIF (10 ng/ml) and/or TGFβ1 (10 ng/ml) for 3 h (**f**–**h**) or 3 days (**i**–**k**) and assayed by QPCR for *Ctgf* (**f**, **i**), *Fn1* (**g**, **j**), and *Col1a1* (**h**, **k**). *N* = 4 technical replicates for each data set. Significant findings were verified with biological replicates of cells sorted from independent donors. * Indicates significantly different from control cultures, # indicates significantly different from TGFβ1 treated cultures, and Φ indicates significantly different from LIF-treated cultures at *P* < 0.05. *P-*values based on ANOVA with Tukey’s multiple comparison test. Source data are provided as a Source Data file

Because FAP-derived fibroblasts are important sources of connective tissue proteins, we assayed whether CD11b/LIF BMT affected FAP numbers in vivo or whether LIF affected the expression of fibrogenic proteins by FAP-derived fibroblasts in vitro. QPCR analysis showed that CD11b/LIF BMT recipients had a 47% reduction in *Pdgfra* expression (Fig. 8a) which could reflect fewer FAPs. Recipients of CD11b/LIF BMT had fewer cells that expressed PDGFRα and were double-negative for CD31 and CD45, which are FAPs^42^ (Fig. 8b, c), although the proportion of PDGFRα+ cells that expressed HSP47 was unaffected by the transgene (Fig. 8d). The findings indicate that reductions in numbers of FAPs in the muscles of *mdx* mice receiving CD11b/LIF BMT may contribute to reduced muscle fibrosis.

We then tested whether LIF influenced the fibrogenic activity of FAP-derived fibroblasts in vitro. We sorted FAPs (CD11b/31/45- PDGFRα+) from WT muscles (Fig. 8e) and subcultured them prior to stimulation with TGFβ1, LIF, or TGFβ1 + LIF^41,42^. We used fibroblasts derived from FAPs rather than freshly-isolated FAPs because fibroblasts differentiated from FAPs are the primary source of connective tissue proteins in muscle^32^. We tested if LIF affected *Pdgfra* expression in fibroblasts in vitro because enhanced PDGFRα signaling can cause pathological fibrosis^43^. However, LIF did not affect *Pdgfra* expression in fibroblasts (mean ± sem: control cells 1 ± 0.04 and LIF-treated cells 1.13 ± 0.23, *n* = 4 per data set, *P* = 0.61; two-tailed *t*-test). Treatments for 3 h with TGFβ1-induced *Ctgf* expression in fibroblasts, but LIF had no effect on basal or TGFβ1-induced *Ctgf* (Fig. 8f). The magnitude of TGFβ1-induced *Ctgf* expression in fibroblasts (1.9-fold) was less than in myoblasts (~11.6-fold) and myotubes (~7.1-fold) (Fig. 7e). TGFβ1, LIF or TGFβ1 + LIF had no effect on *Fn1* or *Col1a1* expression in fibroblasts (Fig. 8g, h). We then tested whether prolonged stimulation of fibroblasts with TGFβ1, LIF, or TGFβ1 + LIF affected *Ctgf*, *Fn1*, or *Col1a1* expression. Similar to effects of brief stimulations, *Ctgf* expression was induced ~2.0-fold by TGFβ1 but the induction was not affected by LIF. There was also no effect of prolonged stimulation with TGFβ1 on the expression of *Fn1* or *Col1a1* (Fig. 8i–k).

### Transplanted CD11b/LIF cells do not affect muscle growth

**Fig. 9:**
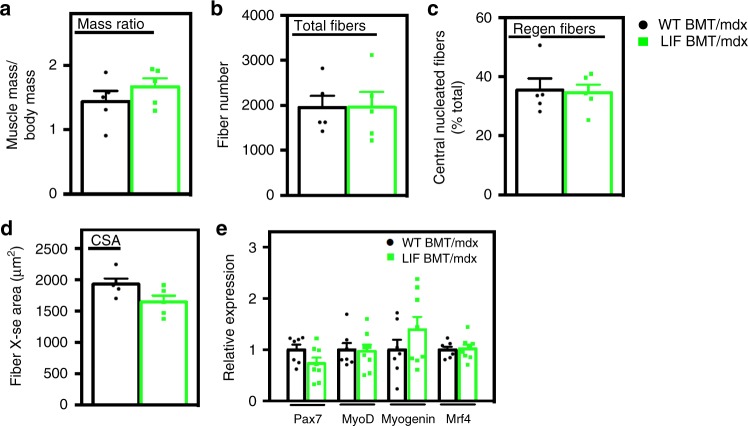
Transplantation of CD11b/LIF transgenic BMCs does not affect muscle growth or regeneration. Assays of muscle mass to body mass ratio (**a**), fiber number (**b**), proportion of centrally-nucleated regenerating fibers (**c**), and muscle fiber cross-sectional area (**d**) indicate no difference in muscle growth or regeneration between WT BMT/*mdx* and LIF BMT/*mdx* recipients. *N* = 5 per group. For all histograms in the figure, the bars indicate mean ± sem. **e** QPCR analysis shows no difference in the expression of myogenic transcription factors (*Pax7*, *Myod1*, *Myog*, and *Mrf4*) in WT BMT/*mdx* versus LIF BMT/*mdx* recipients. *N* = 7 or 8 for WT BMT/*mdx* and LIF BMT/*mdx* data sets, respectively. No significant differences were identified between groups at *P* < 0.05, determined by two-tailed *t-*test. Source data are provided as a Source Data file

Because changes in macrophage phenotype and numbers influence muscle regeneration and myogenesis^14,44–47^, we assayed whether regeneration was affected in CD11b/LIF BMT recipients. There were no significant differences in TA muscle weight, total muscle fiber number, proportions of regenerating fibers or muscle fiber size (Fig. 9a–d). No muscle fibers expressed dMHC in WT BMT/*mdx* or LIF BMT/*mdx* mice. Additionally, QPCR assays showed no effect of CD11b/LIF on expression of the myogenic transcription factors: *Pax7*, *Myod1*, *Myog*, or *Mrf4* (Fig. 9e). These data indicate that the CD11b/LIF transgene did not influence processes through which immune cells modulate regeneration in *mdx* muscle.

## Discussion

The results of our investigation demonstrate that transplantation of genetically-modified BMCs provides a means to deliver therapeutic molecules to dystrophic muscle. In addition, by regulating the expression of the therapeutic transgene with the CD11b promoter, LIF delivery can be modified by the stages of maturation and activation of innate immune cells that differentiate from BMCs. This strategy provides a mechanism for the endogenous regulation of transgene expression by the transplant recipients that is responsive to the magnitude and site of inflammation. This system also permits long-term delivery of therapeutic molecules following a single therapeutic intervention. Although tissues were analyzed 4 months following transplantation in the present investigation, at that time circulating leukocyte populations were nearly 87% donor-derived. However, in humans experiencing BMT, stable mixed chimerism can persist for years in peripheral blood cell populations^48,49^, showing that long-term benefits to humans can result from a single transplantation.

The potential therapeutic advantage of targeting therapeutic molecules to diseased tissue by using transgenes under control of the CD11b promoter is emphasized by comparing our findings with the outcomes of previous strategies to deliver LIF via hematopoietic cell transplantation. Transplantation of a hematopoietic cell line in which the cells were multiply-transduced with a retroviral construct containing cDNA encoding LIF produced high systemic levels of LIF and killed the recipient mice^50,51^. In those experiments the retrovirus-transplant recipients reached serum LIF concentrations at 1400 units/ml, although serum LIF was undetectable in mice transplanted with cells that did not contain the LIF expressing retrovirus^50,51^. This contrasts with the delivery system we employ, in which elevated LIF production was detectible within inflammatory lesions in dystrophic muscle and pathology was reduced, but LIF remained undetectable in the sera. This indicates that more precise temporal and spatial delivery of LIF is necessary for safe and beneficial therapeutic application.

Exogenous LIF has been reported previously to increase the growth of dystrophic muscle fibers^19,20^, but we did not observe an effect of the CD11b/LIF transgene on muscle mass or fiber size in transgenic mice or in CD11b/LIF BMT recipients. These differences in outcome may reflect the different modes of LIF delivery, in which increased fiber size resulted from continuous delivery of high concentrations of exogenous LIF^19,20^. However, we found that transplantation of CD11b/LIF transgenic BMCs affected *mdx* muscle by decreasing muscle fibrosis, consistent with the treatment effect achieved by delivery of exogenous LIF^19,20,52^. In part, the anti-fibrotic influences of the CD11b/LIF transgene were attributable to modifying the phenotype of satellite cells, reflected in the reduced proportion of satellite cells that expressed detectible levels of the collagen chaperone, HSP47, and expressed ERTR7, a connective tissue protein expressed by pro-fibrotic satellite cells^40^. This is functionally important in the context of DMD pathology because the transition of satellite cells from an HSP47−/ERTR7− to an HSP47+/ERTR7+ phenotype reflects a reduction in their myogenic capacity and an increase in their production of connective tissue proteins that may exacerbate the pathology of muscular dystrophy^33^ and lead to a reduction in the regenerative capacity of muscle over time^40^.

Although the CD11b/LIF transgene reduced the expression of pro-fibrotic molecules by muscle cells in CD11b/LIF BMT recipients in vivo, LIF did not reduce the basal level of expression of genes encoding connective tissue proteins by muscle cells in vitro. Instead, we found that LIF reduced the activation of pro-fibrotic genes in myoblasts that was induced by the cytokine TGFβ. TGFβ has broad, profibrotic effects by increasing the expression of major, connective tissue proteins, including collagen and fibronectin^53,54^, and reductions in TGFβ can significantly decrease fibrosis of dystrophin-deficient muscle, at least at early stages of the disease^1,2,29^. In addition to increasing the production of connective tissue proteins, TGFβ can also influence muscle fibrosis by promoting the differentiation of myofibroblasts from muscle^55,56^ and by increasing the expression of other profibrotic growth factors, especially CTGF^53,54^. Our finding that LIF reduced or prevented the TGFβ-mediated induction of *Ctgf* expression in muscle cells may be particularly significant in *mdx* pathology because reductions in *Ctgf* expression can significantly slow pathology^57^. Thus, our in vitro and in vivo data collectively indicate that increases in LIF diminish fibrosis of dystrophic muscle by opposing the profibrotic influence of TGFβ on muscle cells.

The observation that the CD11b/LIF BMT reduced TGFβ1 expression in intramuscular macrophages without causing reductions in total TGFβ1 expression in whole muscle also indicates the specificity of targeting treatment effects that are achieved by the CD11b/LIF transgene. This may provide advantages over other experimental and therapeutic approaches that have been explored previously to reduce fibrosis of dystrophic muscle by inhibiting TGFβ1 expression or activity through pharmacological approaches^1,3,58,59^. While those pharmacological approaches are technically straight-forward and effective at reducing fibrosis in dystrophic muscle, their systemic administration does not provide delivery specifically to sites of inflammation, and increases the risks of off-target effects.

**Fig. 10:**
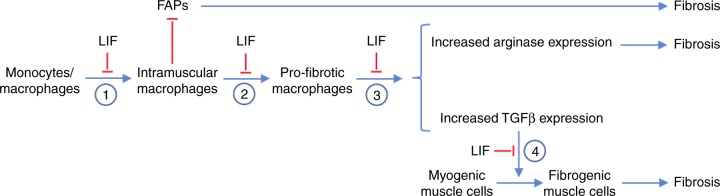
Potential immunomodulatory and anti-fibrotic actions of LIF expressed by the CD11b/LIF transgene in muscular dystrophy. (1) LIF can serve an immunomodulatory role by reducing the expression of *Ccl2* in macrophages, which is associated with reduced recruitment of monocytes/macrophages into dystrophic muscle. (2) LIF can serve an immunomodulatory role by reducing the activation of monocytes/macrophages to a CD163+, M2-biased phenotype that can increase fibrosis of dystrophic muscle. (3) LIF can reduce the expression of the pro-fibrotic molecules *Arg1* and *Tgfb1* in macrophages. (4) LIF can reduce the TGFβ1-mediated induction of pro-fibrotic genes in muscle cells, including *Ctgf* and *Col1a1*

Although CD11b/LIF BMT reduced pathological changes in satellite cells, we found that some beneficial effects of CD11b/LIF transgenic cells are attributable to modulation of the inflammatory response, rather than direct actions on muscle (Fig. 10). Despite the fact that DMD and *mdx* dystrophy result from mutations that cause loss of the membrane-associated structural protein, dystrophin, and lead to a mechanically-weaker muscle cell membrane^60,61^, most muscle fiber damage results from lysis caused by myeloid cells, especially macrophages expressing inducible nitric oxide synthase (iNOS) that are biased toward the M1, pro-inflammatory phenotype^7,26^. However, as the disease progresses, macrophages in dystrophic muscle shift to a CD163+/CD206+ phenotype that increases muscle fibrosis^23^ and is characteristic of type 2 immunity; much of the lethality of DMD is attributable to fibrosis of cardiac and respiratory muscles. Thus, by modulating the numbers and phenotype of macrophages in dystrophic muscle, LIF can produce broad effects on muscle pathology.

Some of the immunomodulatory effects achieved by transplantation of CD11b/LIF transgenic cells reflect the effects of transgene expression within the diseased muscle. For example, *Socs3* expression was significantly elevated in muscles of mice that received CD11b/LIF BMT, although expression of the transgene in macrophages in vitro did not affect the expression of *Socs3*. LIF can increase *Socs3* expression in multiple cell types^62^ and elevated expression or activity of *Socs3* in macrophages can strongly influence their phenotype and cytokine production. In vivo models of inflammation show that siRNA-silencing of SOCS3 or targeted deletion of SOCS3 in macrophages can either promote^63^ or oppose^64^ the M1-biased phenotype. In experimental peritonitis, SOCS3 mRNA silencing in macrophages caused elevated expression of the M2 phenotypic markers *Il10*, *Mrc1*, and *Arg1*^64^, which is consistent with the inverse relationship we observed between elevated *Socs3* expression in CD11b/LIF BMT recipients and reduced expression of *Il10*, *Arg2*, and *Mrc1*. Together, these observations suggest that the shift of CD11b/LIF macrophages away from an M2-biased phenotype in *mdx* BMT recipients may result, in part, from LIF induction of *Socs3* after the transgenic macrophages enter the diseased muscle. However, some of the treatment effects that we observed may have resulted from immunomodulatory roles of the transgene that occurred before their invasion into the pathological muscle. Our finding that isolated BMCs from CD11b/LIF mice showed greatly reduced levels of *Cd163* and *Arg1* expression as they differentiated to macrophages in vitro shows that some autocrine influences of the transgene on macrophage gene expression do not require localization of the cells in the dystrophic muscle. This contrasts with the reduced expression of TGFβ in intramuscular macrophages of CD11b/LIF BMT recipients that did not occur in transgenic macrophages in vitro. The reduction in arginase expression in CD11b/LIF transgenic macrophages may be particularly important in the pathophysiology of muscular dystrophy because arginine metabolism by arginase increases proline production which is necessary for collagen synthesis and contributes significantly to increased fibrosis in *mdx* muscles during progressive stages of pathology^23^.

The immunomodulatory influences of the transgene extend beyond autocrine effects on macrophage phenotype, because the muscles of CD11b/LIF BMC recipients showed large reductions in the expression of ligands for CCR2. Previous investigators established that signaling through CCR2 is a primary mechanism for recruiting macrophages to diseased or injured muscle by showing that blockade or deletion of CCR2 greatly reduces macrophage entry into injured muscle^27,45,47^. We found that CD11b/LIF BMT decreased expression of CCR2 ligands in muscle and reduced the numbers of macrophages that expressed CCL2. Those reductions were also associated with large reductions in total numbers of F4/80+ intramuscular macrophages, including CD206+ and CD163+ macrophages. Thus, much of the anti-inflammatory effect of the transgene may occur through disruption of CCR2-mediated signaling, leading to reduced numbers of intramuscular macrophages and impairing their activation to a pro-fibrotic, M2-biased phenotype.

Collectively, our findings show that expression of a CD11b/LIF transgene in BMDCs can disrupt multiple processes that contribute to fibrosis of dystrophic muscle, including affecting macrophage recruitment, phenotype and production of pro-fibrotic cytokines and enzymes, in addition to preventing the fibrogenic conversion of satellite cells and reducing numbers of FAPs (Fig. 10). However, we believe that the more broadly-significant finding in our investigation is that our data show that genetically-modified BMCs can be used as vectors to deliver therapeutic genes to dystrophic muscle. This approach is applicable not only to LIF, but may provide a more specific targeting strategy for the numerous gene products that have been previously identified as potentially-useful, therapeutic molecules for DMD.

## Methods

### Mice

All experimentation complied with all relevant ethical regulations for animal testing and research, and the study protocol was approved by the Chancellor’s Animal Research Committee at the University of California, Los Angeles. C57BL/10ScSn-Dmd^mdx^/J *mice* (*mdx* mice) were purchased from The Jackson Laboratory (Bar Harbor, ME) and bred in pathogen-free vivaria. *Mdx* mice were selected for use in these experiments instead of more rapidly progressive models of DMD because the goal of our investigation is to test our hypothesis that transplantation of genetically-modified BMCs provides a novel therapeutic strategy for muscular dystrophy. If we used a rapidly, progressive mouse model, such as the *mdx/utr*− mouse line in which the mice die at 2–3 months of age, we would be unable to assay for treatment effects achieved by bone marrow transplantation because the mice would die before enough time passed for sufficient BMC engraftment and then for the transplanted cells to mediate their therapeutic effects.

In preparation for generating CD11b/LIF mice, the complete *Mus musculus* LIF cDNA sequence (611-bp; NM_008501) was amplified by PCR and ligated into a pGL3-Basic vector (Promega) at the Nco I/Xba I sites. The pGL3-Basic vector contained a 550-bp fragment of the human CD11b promoter at the Hind III site, upstream of the LIF insertion site. The 1215-bp, hCD11b/LIF fragment was isolated from pGL3-Basic by restriction endonuclease digestion with Xho I/Xba I and used for pronuclear injection into CB6F1 eggs to generate transgenic mice. Positive founders were identified by PCR screening for presence of the hCD11b/LIF construct and backcrossed with *C57BL/6J* mice for 7 generations. The hCD11b/LIF line is maintained as hemizygous to produce transgenic mice and wild-type, littermate controls for experimentation. Mice were randomly allocated to experimental groups. WT or CD11b/LIF BMCs were transplanted into *mdx* mice assigned non-sequential identification numbers. Investigators collecting data and performing analysis were aware of animal numbers only and were blinded to treatment groups.

CD11b/LIF *mdx* transgenic mice were produced by crossing CD11b/LIF hemizygous males with *mdx* females to generate CD11b/LIF hemizygous, transgenic mice that were also dystrophin-deficient (CD11b/LIF *mdx)*. Dystrophin-deficient status was verified by ARMS PCR screening^65^ and presence of the hCD11b/LIF construct was determined as described above. The CD11b/LIF *mdx* mice were backcrossed with wild-type *mdx* mice for 7 generations to produce CD11b/LIF *mdx* mice that were dystrophin-deficient and either hemizygous or wild-type controls for the CD11b/LIF transgene.

### Bone marrow transplantation

Beginning 1 week prior to BMT, mouse drinking water was supplemented with trimethoprim/sulfamethoxazole (80 μg/ml trimethoprim and 400 μg/ml sulfamethoxazole) and continued for 3 weeks. Two-month-old female *mdx* mice underwent myeloablative preconditioning via intraperitoneal injections of 1,4-butanediol dimethanesulfonate (Sigma-Aldrich) (20 mg/kg body weight) 72-, 48-, and 24-h prior to BMT. On the day of transplantation, male WT and CD11b/LIF donor mice were euthanized and their femur and tibia bones were sterilely dissected and flushed of BMCs. BMCs were isolated and recipient mice received 10^7^ donor BMCs by tail-vein injection. At 4 months post-BMT, tissues and BMCs were collected from recipient mice. BMCs were used for chimerism analysis by fluorescent in situ hybridization of the Y-chromosome (Kreatech FISH Probes).

### RNA isolation and QPCR

RNA was isolated from muscle homogenates and reverse transcribed to produce cDNA^24^. QPCR experiments were designed using established guidelines for experimental design, data normalization and data analysis to maximize the rigor of quantifying the relative levels of mRNA^13,66,67^. The expression for each gene in control samples was set to 1 and the other expression values were then scaled to that value. PCR primers are listed in Supplementary Table 1.

Cultured cells were washed twice with ice-cold DPBS and collected in Trizol (Invitrogen). RNA was extracted and isolated with chloroform extraction and isopropyl alcohol precipitation, followed by clean-up with an RNA Clean and Concentrator Kit (Zymo Research). Total RNA was quantified, reverse transcribed, and used for QPCR^13^.

RNA was isolated from FACS sorted cells by first sorting cells directly into Buffer RLT RNA lysis buffer (Qiagen). RNA was isolated using a Qiagen RNeasy Micro Kit according to the manufacturer’s protocol. RNA yield was quantified using a BioDrop μLite. RNA (50 ng/reaction) was reverse transcribed using a qScript XLT cDNA Supermix (QuantaBio). QPCR experiments were performed on a QuantStudio 5 Real-Time PCR System (Thermo Fisher) with PerfeCTa SYBR Green Supermix, Low Rox (QuantaBio)^13^.

### Immunohistochemistry

Muscles dissected from euthanized mice were frozen in liquid nitrogen-cooled isopentane. Cross-sections 10-µm thick were taken from the mid-belly of muscles and fixed in ice-cold acetone or 2% paraformaldehyde for 10 min. Endogenous peroxidase activity in the sections was quenched by immersion in 0.3% H_2_O_2_. Most sections were blocked for 1 h with blocking buffer (3% bovine serum albumin (BSA), 2% gelatin, and 0.05% Tween-20 in 50 mM Tris–HCl pH 7.6 containing 150 mM NaCl). Alternatively, sections were incubated with 10% horse serum in PBS with 0.1% Tween-20 or mouse-on-mouse blocking reagent (M.O.M. kit; Vector) for sections to be incubated with primary antibodies from goat or mouse origin, respectively. Sections were then incubated with: rat anti-mouse F4/80 (1:100, overnight at 4 °C, eBioscience, clone BM8), rat anti-mouse CD68 (1:100, 3 h at room temperature (RT), AbD Serotec, clone FA-11), rabbit anti-mouse CD163 (1:100, 3 h at RT, Santa Cruz Biotech, clone M-96), rat anti-mouse CD206 (1:50, 3 h at RT, AbD Serotec, clone MR5D3), rat anti-CD4 (1:25, overnight at 4 °C, Biolegend, clone GK1.5), rat anti-Ly-6B.2 (1:25, 2 h at RT, Bio-Rad, clone 7/4), rabbit anti-collagen type 1 (1:50, 3 h at RT, Chemicon, #AB745), goat anti-collagen type 3 (1:50, 3 h at RT, Southern Biotech #1330-01), goat anti-collagen type 5 (1:50, overnight at 4 °C, Southern Biotech, #1350-01), goat anti-LIF (1:66, overnight at 4 °C, R&D Systems, #AB-449), and mouse anti-developmental myosin heavy chain (1:100, overnight at 4 °C, Novacastra, #106304). The sections were washed with phosphate buffered saline (PBS) and probed with biotin-conjugated secondary antibodies (1:200, 30 min at RT, Vector Laboratories). Sections were then washed with PBS and incubated with avidin D-conjugated HRP (1:1000, 30 min at RT, Vector). Staining was visualized with the peroxidase substrate, 3-amino-9-ethylcarbazole (Vector).

### Immunofluorescence

For co-labeling of macrophages, sections were fixed in ice-cold acetone for 10 min and then incubated in blocking buffer for 1 h. Sections were then incubated with rat anti-F4/80 and goat anti-CCL2 (1:50, R&D Systems, AB-479-NA) or rabbit anti-TGFβ1 (1:100, Abcam, #ab92486) overnight at 4 °C. Sections were washed with PBS and then incubated with donkey anti-rat Dylight 488 (1:200, 30 min at RT, Abcam, #ab102260) and horse anti-rabbit IgG Dylight 594 (1:200, 30 min at RT, Vector, #DI-1094) or horse anti-goat IgG Dylight 594 (1:200, 30 min at RT, Vector, #DI-3094). Sections were then washed with PBS and mounted with Prolong Gold mounting media (Invitrogen).

For identification of CCR2+ macrophages, sections were fixed with 4% PFA for 10 min and then incubated with blocking buffer for 1 h. Sections were then labeled with rabbit anti-mouse CCR2 (1:50, Abcam, clone E68) and rat anti-mouse CD68 or rat anti-mouse CD206 at 4 °C overnight. Sections were washed with PBS and then incubated with donkey anti-rat IgG Dylight 594 (1:200, 30 min at RT, Abcam, #ab102262) and horse anti-rabbit IgG Dylight 488 (1:200, 30 min at RT, Vector, #DI-1088).

For identification of fibrogenic satellite cells, sections were fixed in 2% paraformaldehyde for 10 min. Slides were then immersed in antigen retrieval buffer (10 mM sodium citrate, 0.05% Tween-20, pH 6) at 95–100 °C for 40 min, except for sections undergoing Pax7/Ertr7 co-labeling this step was omitted. Sections were then treated with blocking buffer from a mouse-on-mouse immunohistochemistry kit (M.O.M. kit; Vector) for 1 h and immunolabeled with mouse anti-Pax7 and rabbit anti-HSP47 (1:200, Abcam, #77609) or anti-Ertr7 (1:1000, SCBT, #SC-73355) overnight at 4 °C. Anti-Pax7 was purified from hybridoma cell supernatant (Developmental Studies Hybridoma Bank, Iowa City, Iowa)^68^. Sections were washed with PBS and then incubated with horse anti-mouse IgG Dylight 594 (1:200, 30 min at RT, Vector, DI-2594) and horse anti-rabbit IgG Dylight 488 (1:200, 30 min at RT).

For identification of FAPs, sections were fixed in ice-cold acetone for 10 min and then incubated with blocking buffer for 1 h. Sections were then labeled with rat anti-mouse CD31 conjugated with FITC (1:50, eBioscience, clone 390), rat anti-mouse CD45 conjugated with FITC (1:100, eBioscience, clone 30-F11), and goat anti-PDGFRα (1:100, R&D Systems, #AF1062) at 4 °C overnight. Sections were washed with PBS and then incubated with horse anti-goat IgG Dylight 594 (1:200, 30 min at RT, Vector).

For identification of fibrogenic PDGFRα+ cells, sections were fixed in ice-cold acetone for 10 min and then incubated with blocking buffer for 1 h. Sections were then labeled with rabbit anti-mouse HSP47 (1:100, ABcam, clone EPR4217) and goat anti-PDGFRα at 4 °C overnight. Sections were washed with PBS and then incubated with horse anti-goat IgG Dylight 594 (1:200, 30 min at RT) and horse anti-rabbit IgG Dylight 488 (1:200, 30 min at RT).

### Stereology

The number of cells per volume of muscle was determined by measuring the total volume of each section using a stereological, point-counting technique to determine section area and then multiplying that value by the section thickness (10 μm)^7^. The numbers of immunolabeled cells in each section were counted and expressed as the number of cells per unit volume of each section.

### Assays for fiber number, central nucleation, and size

TA muscles were sectioned at the midbelly of muscles and used for fiber cross-sectional area measurements^5^. The proportion of fibers containing central nuclei, an indicator of fiber regeneration, was also determined. Central-nucleation was expressed as the ratio of central nucleated fibers relative to the entire population of fibers sampled for each muscle. The cross-sectional areas of >300 muscle fibers were measured using a digital imaging system (BioQuant).

### Assay of muscle connective tissue content

The volume fraction of muscle that was occupied by collagen types 1, 3, and 5 was determined by overlaying a 10 × 10 eye-piece grid on microscopic images of cross-sections of entire muscle that were immunolabeled with antibodies to collagen types 1, 3, or 5.

### Preparation of BMMCs and BMDMs

BMMCs were separated from whole BMC preparations flushed from WT or CD11b/LIF femurs and tibiae and separated using a histopaque-1077 gradient (Sigma). The freshly-isolated BMMCs were then used for RNA isolation and analysis. For preparation of BMDMs, BMCs were aseptically flushed from WT or CD11b/LIF femurs and tibiae and differentiated in vitro to BMDMs^24^. BMDMs were stimulated for 24 h with activation media consisting of Dulbecco’s Modified Eagle Medium (DMEM) with 0.25% heat-inactivated fetal bovine serum (FBS; Omega), 100 U/ml penicillin, 100 µg/ml streptomycin (1% P/S), and 10 ng/ml macrophage colony stimulating factor (MCSF; R&D).

### ELISA analysis of BMDM conditioned media

Cultures of BMDM from WT mice were established as described above. On the 6th day of culture, the BMDMs were switched to DMEM containing 1% P/S, 0.25% heat-inactivated FBS, and M-CSF or media containing 10 ng/ml recombinant mouse LIF (eBioscience) (stimulation media). After 24 h of stimulation, conditioned media (CM) were collected, briefly centrifuged to remove particulates and then frozen at −20 °C. Separate aliquots of BMDM CM from each sample were analyzed for expression of CCL2 (Duoset ELISA, R&D Systems, #DY479) and TGFβ (Emax immunoassay; Promega, #G7590), according to manufacturer’s instructions. Following an HRP-based reaction, a colored product was formed in proportion to the amount of cytokine present, which was analyzed by a spectrophotometer (Bio-Rad Benchmark Microplate Reader) at a wavelength of 450 nm with a 540 nm correction. The concentration of each cytokine was determined by comparing the optical density of the samples to the standard curve.

### ELISA analysis of serum

Whole blood was collected from the femoral artery and allowed to clot on ice for at least 30 min. The whole blood was spun for 10 min at 2000×*g* at 4 °C. The serum was collected and stored in liquid nitrogen until analyzed for circulating LIF, TNFα, IFNγ, IL-4, and IL-10 by ELISA, according to manufacturer’s instructions (R&D Systems, Quantikine ELISAs, #MLF00, MTA00B, MIF00, M4000B, and M1000B). Each group contained 3 replicates.

### Assays for LIF effects on muscle cell fibrogenesis

The C2C12 cell line was purchased from American Type Culture Collection (ATCC CRL-1772 cell line). The cells were authenticated as myoblasts by confirming their differentiation into contractile myotubes that express characteristic muscle proteins. Cells were seeded in six-well plates at 6 × 10^4^ cells per well and cultured in DMEM containing 10% FBS, 1% P/S at 37 °C in 5% CO_2_ for 24 h and then serum-starved overnight prior to stimulation. To generate myotubes, myoblasts were grown to 90% confluence and then differentiated in serum-free DMEM for 24 h. The cells were then returned to DMEM containing 10% FBS for 5 days. Myoblast and myotube cultures were stimulated with vehicle, TGFβ1 (10 ng/ml), LIF (10 ng/ml), or TGFβ1 + LIF for 3- or 24-h.

### FAPs and myogenic progenitor cell preparation and isolation

FAPs were isolated from 6-month-old WT mice. Hindlimb and forelimb muscles were dissected and rinsed in DMEM. Muscles were minced and digested in 5 ml of enzyme buffer (DMEM, 25 mM HEPES (Sigma), 5 mM MgCl_2_ (Fisher), 2% P/S, 12.5 U dispase, type II, 12.5 U collagenase B, and 20 μg/ml DNase I (Roche)) for 60 min at 37 °C with gentle trituration. The digestion was neutralized with 2 volumes of staining buffer (DMEM, 10 mM NaHCO_3_ (EMD Millipore), 25 mM HEPES, 5 mM EDTA, 5 mM MgCl_2_, 1 mM L-glutamine, 2% BSA, and 2% P/S). The digestate was passed through 100 μm mesh filters and cells were pelleted at 350*g* for 5 min. Cells were resuspended in ACK lysis buffer (Lonza) for 5 min followed by the addition of an equal volume of staining buffer and cells were pelleted at 350*g* for 5 min. Cells were resuspended in staining buffer with CD16/32 (eBioscience #14-0161-85; 0.5 µg/test) for 10 min to block Fc receptor binding. Cells were labeled with Hoechst (Sigma #14533) and antibodies against CD11b (eBioscience #11-0112; 0.25 µg/test), CD31 (eBioscience #11-0311; 0.5 µg/test) and CD45 (eBioscience #11-0451; 0.1 µg/test) conjugated with FITC and PDGFRα conjugated with PE-Cy7 (eBioscience #25-1401; 0.2 µg/test). FAPs (Hoechst + CD11b/31/45-PDGFRα+) were sorted into collection buffer (DMEM, 10 mM NaHCO_3_ and 20% FBS) using a BD SORP FACSAriaII cell sorter.

MPCs were isolated from 14-months-old CD11b/LIF *mdx* mice and littermate controls. Hindlimb and forelimb muscles were dissected and digested as described for FAPs isolation. Isolated cells were resuspended in staining buffer with CD16/32 for 10 min to block Fc receptor binding. Cells were labeled with cell impermeant dye DAPI (Sigma) to distinguish live cells and antibodies against CD11b, CD31, and CD45 conjugated with FITC and Sca-1 conjugated with PE-Cy5 (eBioscience #15-5981; 0.2 µg/test), integrin α7 conjugated with PE (Medical and Biological Laboratories #K0046-5; 15 µl/test). Live MPCs (DAPI-CD11b/31/45-Sca1-α7int+) were sorted into Buffer RLT RNA lysis buffer (Qiagen) using a FACSAriaIII high speed cell sorter.

### Primary fibroblast cell culture

Sorted FAPs were cultured in growth medium (DMEM, 20% FBS, 10% heat-inactivated horse serum, 1% P/S and 2.5 ng/ml bFGF) on tissue culture plates coated with Matrigel^41^. After plating, cells were cultured for 3 days and half the medium was changed. Cells were expanded and subcultured. Prior to stimulation, cells were cultured in reduced serum media overnight (DMEM, 2% FBS, 1% P/S, and 2.5 ng/ml bFGF). Fibrogenic cell cultures were stimulated with vehicle, TGFβ1 (10 ng/ml), LIF (10 ng/ml), or TGFβ1 + LIF for 3 h or 3 days (with media changes at 24- and 48-h).

### Physiological analysis

We assayed muscle stiffness and viscoelasticity at 14 months of age because connective tissue accumulation in *mdx* muscle is progressive between 3 and 24 months of age. We expected that if we sampled for effects of the transgene on muscle stiffness during the late, progressive stage of the disease, the magnitude of the effect would be greater, which would enable us to address more definitively the question of whether the transgene influenced muscle stiffness. Male WT/*mdx* and LIF/*mdx* mice were anesthetized by the intraperitoneal (i.p.) injection of ketamine (40 mg/kg body weight). Anesthesia was checked by testing mice for a positive reflex response to a hind foot pinch and by monitoring respiration. Additional i.p. injections of ketamine were given throughout the study, as needed. For in-situ analysis of the TA muscle the knee was immobilized to the heated (37 °C) platform of an 809C in-situ mouse apparatus (Aurora Scientific). Silk sutures (5-0; Ethicon) were knotted to the distal, severed tendon and then secured to the lever arm of a dual-mode force transducer-servomotor (Aurora Scientific, Model 305C-5N). After placing platinum-tipped electrodes into the leg above the knee, flanking the sciatic nerve, the TA muscle was stimulated by pulses and manipulated on three axes to find the optimal muscle length (*L*_o_). *L*_o_ was multiplied by the pennation of 0.6 for the TA muscle^69^ to determine optimal fiber length (*L*_f_). To measure elasticity, the muscle was left unstimulated while the lever arm oscillated at ±20% of the *L*_f_ for 20 s. Muscles were allowed to rest for 60 s before subsequent oscillation series. Muscles were allowed to rest for 60 s before a series of oscillations at 3 Hz, which provides a physiological strain and strain rate^70^. Dynamic Muscle Control and Dynamic Muscle Analysis (Aurora Scientific) software was used to conduct experiments and record data. Force measurements were normalized to muscle cross-sectional area, and position measurements were normalized to percent of *L*_f_.

### Statistical analyses

All data are presented as mean ± sem. Statistical significance was calculated using unpaired Student’s *t-*tests or ordinary one-way ANOVA with Tukey’s multiple comparison test to determine differences among multiple groups. Differences with a *P-*value < 0.05 were considered statistically significant. The equality of variance between the groups that are being compared was tested with an *F* test, experiments with a *P-*value < 0.05 are denoted in the figure legend. Additionally, for immunohistochemistry and immunofluorescence experiments, slides were only included if concurrently immunolabeled. Statistical analysis was performed using GraphPad Prism.

### Reporting summary

Further information on research design is available in the Nature Research Reporting Summary linked to this article.

## Supplementary Information

Supplementary Information

Reporting Summary

Source data

## Data Availability

The authors declare that the data supporting the findings of this study are available within the paper and its Supplementary Information files. The source data pertaining to Figs. 1a–c, 2a, d, f, g, i, j, m, p–r, 3a, b, e, h, k, l, 4a–f, h, 5g, j, k, 6a, d, e, 7c–f, 9a–d, f and Supplementary Figs. 1a, 1b, 1c, 1d, 2a, 2b, 3a, 3b, 3c, 3d, 4a, 4b, 4c, 5b, 5c, 5d, 5e, and 5f are provided as a Source Data file.
