# Supplementary material for: Targeting a therapeutic LIF transgene to muscle via the immune system ameliorates muscular dystrophy
Source: Nat Commun. 2019 Jun 26;10:2788. doi: 10.1038/s41467-019-10614-1 (PMC6594976; doi:10.1038/s41467-019-10614-1)
Supplement: Supplementary file 1 — Supplementary Information [file 41467_2019_10614_MOESM1_ESM.pdf]

Supplementary information

Targeting a therapeutic LIF transgene to muscle via the immune system ameliorates muscular dystrophy.

Welc et al.

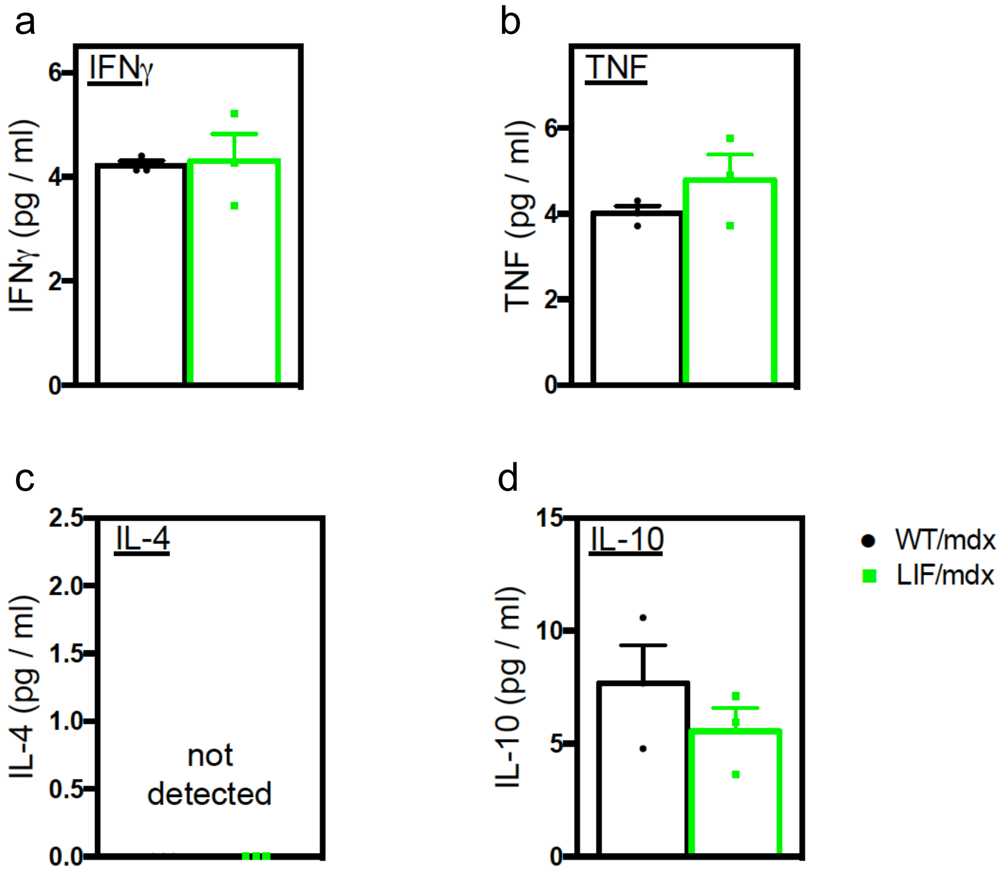

Supplementary figure 1: Expression of a CD11b/LIF transgene in *mdx mice* does not affect systemic pro-inflammatory or anti-inflammatory cytokine expression in *mdx mice*. Serum ELISA for circulating levels of IFNγ (A), TNF (B), IL-4 (C) and IL-10 (D) showed no significant change in serum cytokine concentrations of 3-months old WT/*mdx* and LIF/*mdx mice*. For all histograms in the figure, the bars indicate mean ± *sem*. N = 3 for each data set. No significant differences were identified between groups using *P*-values < 0.05, determined by two-tailed *t*-test. Source data are provided as a Source Data file.

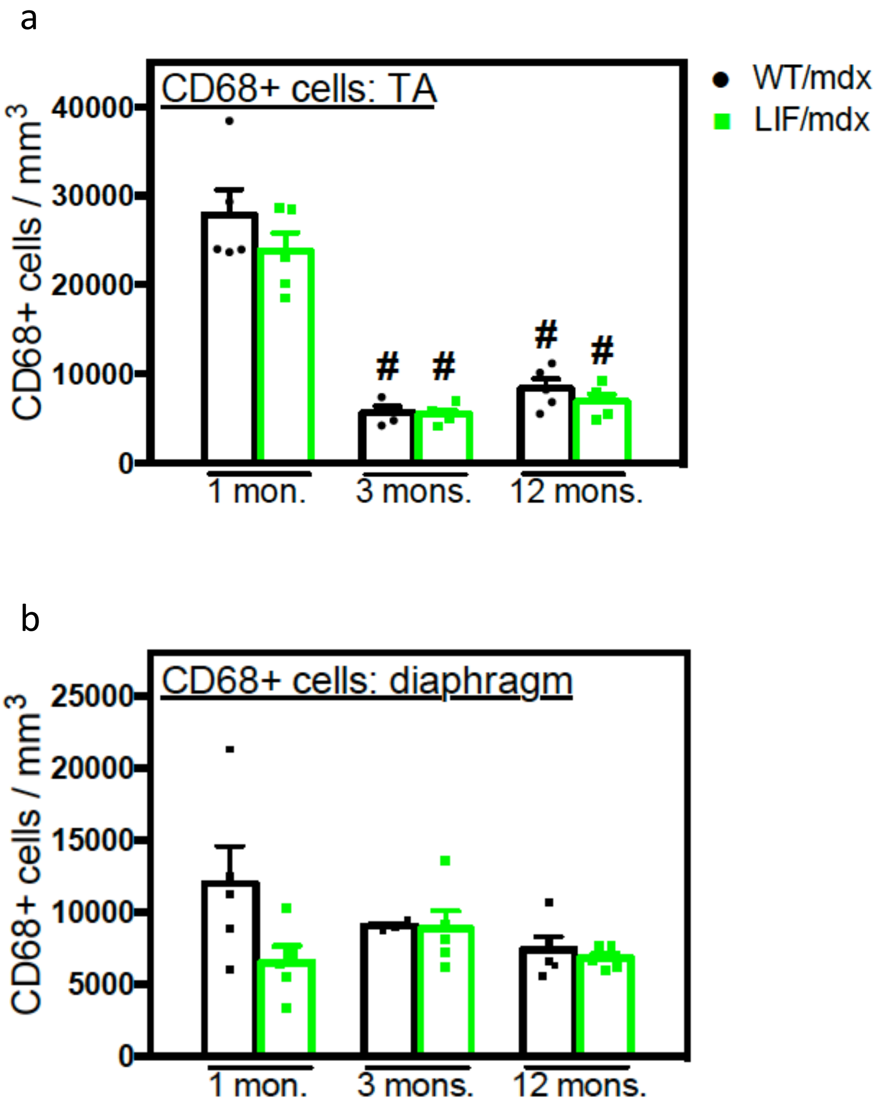

Supplementary figure 2: Expression of a CD11b/LIF transgene in *mdx mice* does not affect CD68+ cell numbers in TA or diaphragm muscle. TA (A) and diaphragm (B) muscles of WT/*mdx* and LIF/*mdx* transgenic *mice* were immunolabeled and the numbers of CD68+ cells at the ages of 1-, 3- and 12-months were quantified. No significant differences in the number of CD68+ cells were found between genotypes at the ages tested. For all histograms in the figure, the bars indicate mean ± *sem*. N = 5 for each data set, except n = 4 for 3-months WT/*mdx mice*. # indicates significant difference versus 1-month *mice* of the same genotype at $P < 0.05$. *P*-values based on two-tailed *t*-test. Source data are provided as a Source Data file.

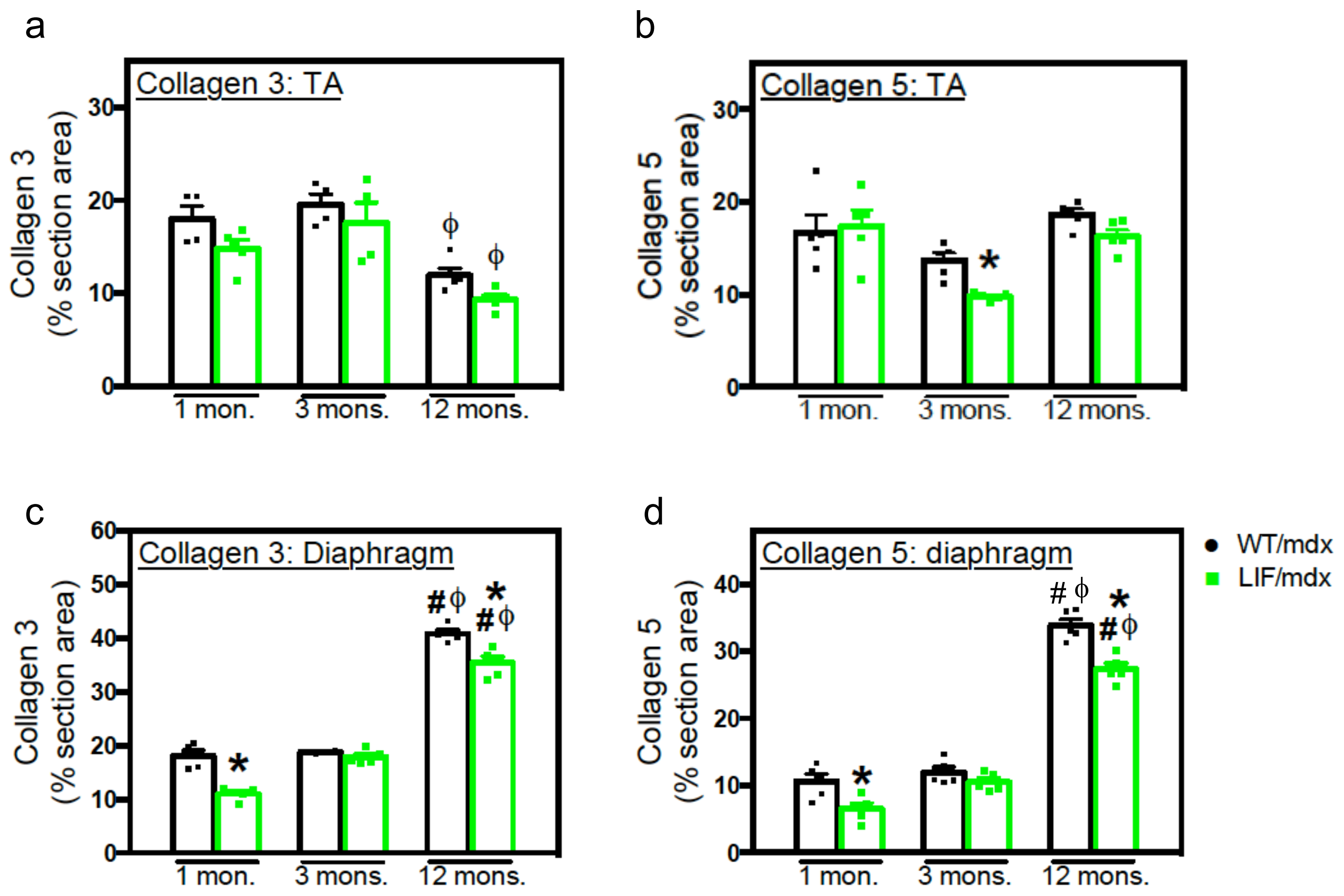

Supplementary figure 3: Expression of a CD11b/LIF transgene in *mdx mice* reduces collagen types 3 and 5 accumulation in TA and diaphragm muscles. (A-D) TA and diaphragm muscles of WT/*mdx* and LIF/*mdx mice* were immunolabeled for collagen types 3 (A, C) or 5 (B, D) at the ages of 1-, 3- and 12-months. The volume fraction of area occupied by each collagen type was quantified in the TA (A, B) and diaphragm (C, D). The accumulation of collagen type 3 was reduced in 1- and 12-months diaphragms of LIF/*mdx mice*. Collagen type 5 accumulation was also reduced in 3-months TAs, 1- and 12-months diaphragms of LIF/*mdx mice*. For all histograms in the figure, the bars indicate mean ± *sem*. N = 5 for each data set, except n = 4 for collagen type 3 WT/*mdx* 1- and 3-month TA, LIF/*mdx* 3-month TA, WT/*mdx* 3-month diaphragm data sets. * indicates significant difference versus WT/*mdx mice* of the same age at $P < 0.05$. # indicates significant difference versus 1-month *mice* of the same genotype at $P < 0.05$. Φ indicates significant difference versus 3-months-old *mice* of the same genotype at $P < 0.05$. *P*-values based on two-tailed *t*-test. *F*-test collagen type 3 3-month diaphragm ($P = 0.0242$) and collagen type 5 3-month TA ($P = 0.0139$). Source data are provided as a Source Data file.

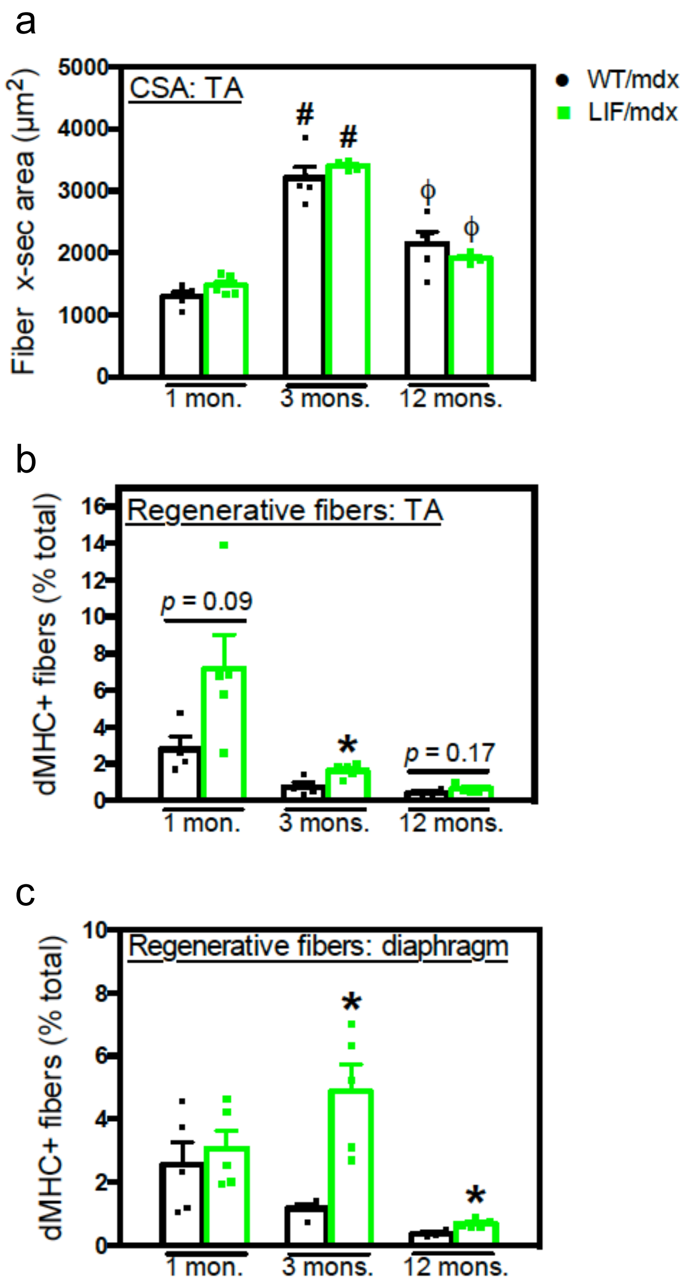

Supplementary figure 4: Expression of a CD11b/LIF transgene in *mdx mice* does not affect muscle fiber growth but increases the formation of regenerating fibers. (A) The average fiber cross-sectional area of WT/*mdx* and LIF/*mdx* TAs was quantified at 1-, 3- and 12-months. No significant differences were detected between the two genotypes at the ages tested. For all histograms in the figure, the bars indicate mean ± *sem*. N = 5 for each data set, except n = 4 for 3- and 12-months LIF/*mdx* TA muscles. # indicates significant difference versus 1-month *mice* of the same genotype at $P < 0.05$. Φ indicates significant difference versus 3-months-old *mice* of the same genotype at $P < 0.05$. *P*-values based on two-tailed *t*-test. *F*-test TA muscle fiber cross-sectional area 3- ($P = 0.0222$) and 12-months TA ($P = 0.0230$). (B-C) TA and diaphragm muscles of WT/*mdx* and LIF/*mdx mice* were immunolabeled with antibodies to developmental myosin heavy chain (dMHC) at 1-, 3- and 12-months. The proportion of dMHC+ to total muscle fibers was quantified. The proportion of dMHC+ fibers increased in TA muscles at 3-months (B) and diaphragm muscles at 3- and 12-months (C). Data are presented as mean ± *sem*, n = 5 for each data set, except n = 4 for 1-, 3- and 12-months WT/*mdx* TA muscles and 3- and 12-months WT/*mdx* diaphragm muscles. * indicates significant difference versus WT/*mdx mice* of the same age at $P < 0.05$. *P*-values based on two-tailed *t*-test. *F*-test diaphragm muscle fiber cross-sectional area 3-months ($P = 0.0113$). Source data are provided as a Source Data file.

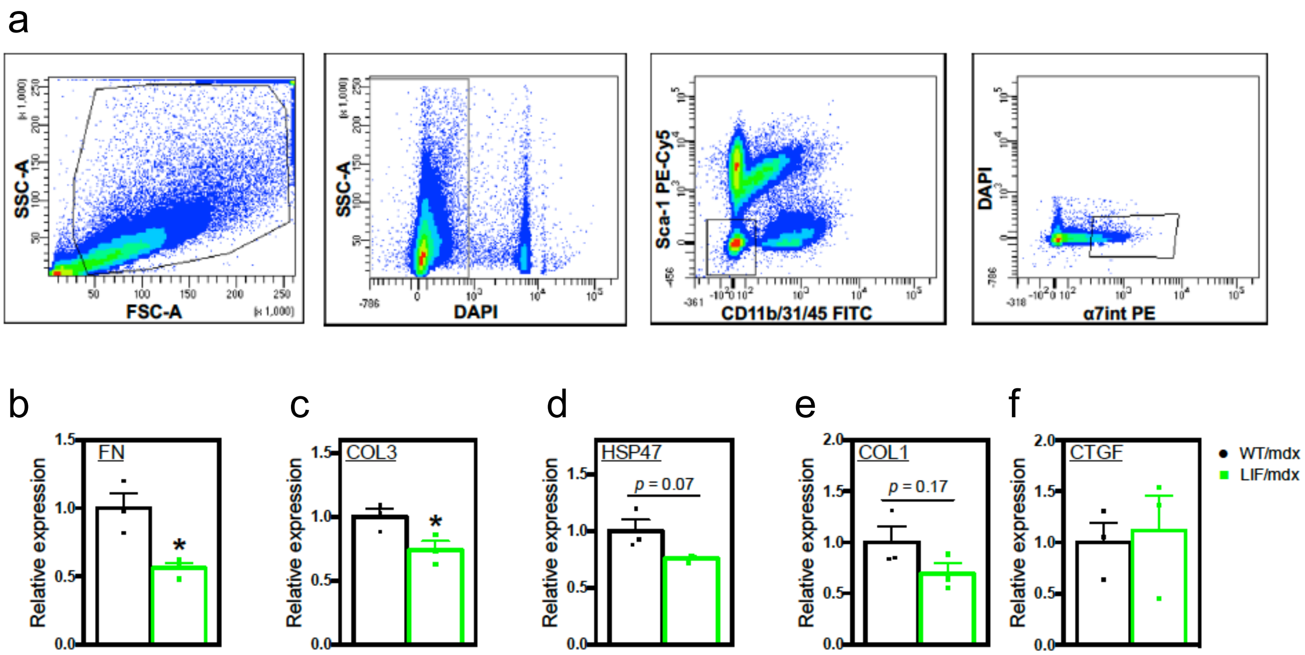

Supplementary figure 5: Expression of a CD11b/LIF transgene in *mdx mice* attenuates expression of fibrogenic genes in myogenic progenitor cells. (A) FACS plots demonstrating strategy for sorting myogenic progenitor cells (DAPI-CD11b-CD31-CD45-Sca1-Intα7+) from 14-months old WT/*mdx* and LIF/*mdx mice*. (B-F) RNA was collected from sorted myogenic progenitor cells and used for QPCR analysis of *Fn* (B), *Col3a1* (C), *Hsp47* (D), *Col1a1* (E) and *Ctgf* (F). For all histograms in the figure, the bars indicate mean ± *sem*. N = 3 for each data set. * indicates significant difference versus WT/*mdx mice* at $P < 0.05$. *P*-values based on two-tailed *t*-test. Source data are provided as a Source Data file.

Gene	Forward	Reverse
Cd11b	CATGAATGATGCTTACCTGGGTTATG	CCCAAAATAAGAGCCAATCTGG
Lif	GTCTTGGCCGCAGGGATTG	GCACAGGTGGCATTTACAGG
Serpinh1	GACCCATGACCTGCAGAAAC	GAAGGCAGTGGCATGGAAC
Cd68	CAAAGCTTCTGCTGTGGAAAT	GACTGGTCACGGTTGCAAG
iNOS	CAGCACAGGAAATGTTTCAGC	TAGCCAGCGTACCGGATGA
Cd163	GCAAAAACTGGCAGTGGG	GTCAAAATCACAGACGGAG
Cd206	GGATTGTGGAGCAGATGGAAG	CTTGAATGGAAATGCACAGAC
Arg1	CAATGAAGAGCTGGCTGGTGT	GTGTGAGCATCCACCCAAATG
Arg2	GAAGTGGTTAGTAGAGCTGTGTC	GGTGAGAGGTGTATTAATGTCCG
Tnf	CTTCTGTCTACTGAACTTCGGG	CACTTGGTGGTTTGCTACGAC
Ifng	GACAATCAGGCCATCAGCAAC	CGGATGAGCTCATTGAATGCTT
Il1b	GTAATGAAAGACGGCACACC	CTCTGCAGACTCAAACTCC
Il6	GAACAACGATGATGCACTTGC	CTTCATGTACTCCAGGTAGCTATGGT
Il12a	TGCCTTGGTAGCATCTATGAG	TTCAGGCGGAGCTCAGATAG
Il4	GGATGTGCCAAACGTCCTC	GAGTTCTTCTTCAAGCATGGAG
Il10	CAAGGAGCATTTGAATTCCC	GGCCTTGTAGACACCTTGGTC
Tgfb1	CTCCACCTGCAAGACCAT	CTTAGTTTGGACAGGATCTGG
Socs3	CTTTCTTATCCGCGACAGCTC	CACTGGATGCGTAGGTTCTTG
Ccr2	CCTGTAAATGCCATGCAAGTTC	GTATGCCGTGGATGAACTGAG
Ccl2	GCTCAGCCAGATGCAGTTAAC	CTCTCTCTTGAGCTTGGTGAC
Ccl7	CAACCAGATGGGCCCAATG	GATAACAGCTTCCCAGGGACAC
Ccl8	GATAAGGCTCCAGTCACCTGC	CCCTGCTTGGTCTGGAAAAC
Ccl12	CTGGACCAGATGCGGTGAG	AAGATCACAGCTTCCCGGG
Col1a1	TGTGTGCGATGACGTGCAAT	GGGTCCCTCGACTCCTACA
Col3a1	ATCCCATTTGGAGAATGTTGTGC	GGACATGATTCACAGATTCCAGG
Col5a3	CGGGGTACTCCTGGTCCTAC	GCATCCCTACTTCCCCCTTG
Axin2	GACGCACTGACCGACGATTC	CTGCGATGCATCTCTCTCTGG
Ctgf	GGACACCTAAAATCGCCAAGC	GGCACAGGTCTTGATGAACATC
Fn	GCTCAGCAAATCGTGCAGC	CTAGGTAGGTCCGTTCCCACTG
Snai1	CTTGTGTCTGCACGACCTGTG	GTCAGCAAAAGCACGGTTG
Tpt1	GGAGGGCAAGATGGTCAGTAG	CGGTGACTACTGTGCTTTCG
Rnps1	AGGCTCACCAGGAATGTGAC	CTTGGCCATCAATTTGTCCT
Rpl13a	CCTGCTGCTCTCAAGGTTGTT	CGATAGTGCATCTTGGCCTTT
Srp14	AGAGCGAGCAGTTCCTGAC	CGGTGCTGATCTTCCTTTTC
Rplp0	GGACCCGAGAAGACCTCCTT	GCTGCCGTTGTCAAACACC

Supplementary Table 1: Primer sequences.
