# Supplementary material for: Targeting a therapeutic LIF transgene to muscle via the immune system ameliorates muscular dystrophy
Source: Nat Commun. 2019 Jun 26;10:2788. doi: 10.1038/s41467-019-10614-1 (PMC6594976; doi:10.1038/s41467-019-10614-1)
Supplement: Supplementary file 2 — Reporting Summary [file 41467_2019_10614_MOESM2_ESM.pdf]

nature research

Corresponding author(s): James G. Tidball

Last updated by author(s): May 1, 2019

Reporting Summary

Nature Research wishes to improve the reproducibility of the work that we publish. This form provides structure for consistency and transparency in reporting. For further information on Nature Research policies, see Authors & Referees and the Editorial Policy Checklist.

Statistics

For all statistical analyses, confirm that the following items are present in the figure legend, table legend, main text, or Methods section.

n/a	Confirmed	
☐	☒	The exact sample size (*n*) for each experimental group/condition, given as a discrete number and unit of measurement
☐	☒	A statement on whether measurements were taken from distinct samples or whether the same sample was measured repeatedly
☐	☒	The statistical test(s) used AND whether they are one- or two-sided<br>*Only common tests should be described solely by name; describe more complex techniques in the Methods section.*
☒	☐	A description of all covariates tested
☐	☒	A description of any assumptions or corrections, such as tests of normality and adjustment for multiple comparisons
☐	☒	A full description of the statistical parameters including central tendency (e.g. means) or other basic estimates (e.g. regression coefficient) AND variation (e.g. standard deviation) or associated estimates of uncertainty (e.g. confidence intervals)
☐	☒	For null hypothesis testing, the test statistic (e.g. *F*, *t*, *r*) with confidence intervals, effect sizes, degrees of freedom and *P* value noted<br>*Give P values as exact values whenever suitable.*
☒	☐	For Bayesian analysis, information on the choice of priors and Markov chain Monte Carlo settings
☒	☐	For hierarchical and complex designs, identification of the appropriate level for tests and full reporting of outcomes
☒	☐	Estimates of effect sizes (e.g. Cohen's *d*, Pearson's *r*), indicating how they were calculated

Our web collection on statistics for biologists contains articles on many of the points above.

Software and code

Policy information about availability of computer code

Data collection — *Provide a description of all commercial, open source and custom code used to collect the data in this study, specifying the version used OR state that no software was used.*

Data analysis — *Provide a description of all commercial, open source and custom code used to analyse the data in this study, specifying the version used OR state that no software was used.*

For manuscripts utilizing custom algorithms or software that are central to the research but not yet described in published literature, software must be made available to editors/reviewers. We strongly encourage code deposition in a community repository (e.g. GitHub). See the Nature Research guidelines for submitting code & software for further information.

Data

Policy information about availability of data

All manuscripts must include a data availability statement. This statement should provide the following information, where applicable:

- Accession codes, unique identifiers, or web links for publicly available datasets
- A list of figures that have associated raw data
- A description of any restrictions on data availability

The authors declare that the data supporting the findings of this study are available within the paper and its supplementary information files. "The source data pertaining to Figures 1a, 1b, 1c, 2a, 2d, 2f, 2g, 2i, 2j, 2m, 2p, 2q, 2r, 3a, 3b, 3e, 3h, 3k, 3l, 4a, 4b, 4c, 4d, 4e, 4f, 4h, 5g, 5j, 5k, 6a, 6d, 6e, 7c, 7d, 7e, 7f, 9a, 9b, 9c, 9d, 9f and Supplementary Figures 1a, 1b, 1c, 1d, 2a, 2b, 3a, 3b, 3c, 3d, 4a, 4b, 4c, 5b, 5c, 5d, 5e and 5f are provided as a Source Data file.

Field-specific reporting

Please select the one below that is the best fit for your research. If you are not sure, read the appropriate sections before making your selection.

☒ Life sciences ☐ Behavioural & social sciences ☐ Ecological, evolutionary & environmental sciences

For a reference copy of the document with all sections, see nature.com/documents/nr-reporting-summary-flat.pdf

Life sciences study design

All studies must disclose on these points even when the disclosure is negative.

Sample size	The initial experimental design was formulated based on our extensive experience using genetically modified mice to study the inflammatory response in mdx dystrophy. We were unable to do an a priori power analysis for our experiments because the experiments relied on novel experimental treatments; thus we did not know the magnitude of the effect size needed for a priori power analysis. Additionally, a detailed description of the post-hoc statistical analyses used in this study have been provided to determine differences between groups.
Data exclusions	Data sets of ≥ 5 were analyzed for outliers using Grubbs' outlier (P<0.05) test to determine if one of the values in each data set is a statistical outlier from rest of the values. Outliers were tested and removed prior to statistical comparisons between groups.
Replication	To verify reproducibility of our findings, tissue samples in each data set came from distinct animals. In vitro cell culture findings were assayed first by using technical replicates to test for treatment effects. Then, the reproducibility of significant findings was verified by performing a separate, independent experiment.
Randomization	All mice and cell culture samples were randomly allocated into experimental groups.
Blinding	Mice were assigned non-sequential identification numbers. Investigators collecting data and performing analysis were aware of animal numbers only and were blinded to treatment groups.

Reporting for specific materials, systems and methods

We require information from authors about some types of materials, experimental systems and methods used in many studies. Here, indicate whether each material, system or method listed is relevant to your study. If you are not sure if a list item applies to your research, read the appropriate section before selecting a response.

Materials & experimental systems

n/a	Involved in the study	
☐	☒	Antibodies
☐	☒	Eukaryotic cell lines
☒	☐	Palaeontology
☐	☒	Animals and other organisms
☒	☐	Human research participants
☒	☐	Clinical data

Methods

n/a	Involved in the study	
☒	☐	ChIP-seq
☒	☐	Flow cytometry
☒	☐	MRI-based neuroimaging

Antibodies

Antibodies used

Rat anti-mouse F4/80; eBioscience #14-4801; Clone BM8; Lot: E04273-1636.
Rat anti-mouse CD68; AbD Serotec #MCA1957; Clone FA-11; Lot: 0114.
Rabbit anti-mouse CD163; Santa Cruz Biotech #sc-33560; Clone M-96; Lot: E1614.
Rat anti-mouse CD206; AbD Serotec #MCA2235; Clone MR5D3; Lot: 0311.
Rat anti-mouse CD4; Biolegend #100401; Clone GK1.5.
Rat anti-mouse Ly6B.2; Biorad #MCD771G; Clone 7/4.
Rabbit anti-human Collagen type 1; Chemicon #AB745; Lot: 102088D1.
Goat anti- Collagen type 3; Southern Biotech #1330; Lot: A4611-5356.
Goat anti- Collagen type 5; Southern Biotech #1350; Lot: F5302-5576.
Goat anti- mouse Leukemia Inhibitory Factor; R&D Systems #AB-449; Lot: GP044031.
Mouse anti- human developmental myosin heavy chain; Novocastra #106304; Clone RNMY2/9D2.
Goat anti- mouse CCL2; R&D Systems #AB-479-NA; Lot: OJ0311061.
Rabbit anti-mouse TGF beta; Abcam #ab92486; Lot: GR312172-2.
Rabbit anti- mouse CCR2; Abcam #ab32144; Lot: GR52022-2.
Mouse anti- mouse Pax7; Developmental Studies Hybridoma Bank product PAX7.
Rabbit anti- mouse HSP47; Abcam #ab77609; Lot: GR22989-2.
Rat anti- mouse Ertr7; Santa Cruz #sc-73355; Lot: F1113.
Rat anti- mouse CD31; eBioscience #11-0311; Clone 390; Lot: 4291915.
Rat anti- mouse CD45; eBioscience #11-0451; Clone 30-F11; Lot: 4277450.

	Rat anti- mouse CD11b; eBioscience #11-0112; Clone M1/70; Lot: E00148-1634.<br>Goat anti- mouse PDGFR alpha; R&D Systems #AF1062; Lot: HMQ0215021.<br>Rat anti- mouse CD16/32; eBioscience #14-0161; Clone 93; Lot: E03558-1633.<br>Rat anti- mouse PDGFR alpha; eBioscience #25-1401; Clone APA5; Lot: 4274748.<br>Rat anti- mouse Sca-1; eBioscience #15-5981; Clone D7; Lot: E06259-1633.<br>Mouse anti- mouse integrin alpha 7; Medical and Biological Laboratories #K0046-5; Clone 3c12.
Validation	Rat anti-mouse F4/80; eBioscience #14-4801; Specificity tested by manufacturer using flow cytometry; Our standard laboratory procedure included the use of no primary antibody reagent control to ensure the staining is produced from the detection of the antigen by the primary antibody and not by the tissue specimen or reagent detection system.<br>Rat anti-mouse CD68; AbD Serotec #MCA1957; Antibody validated for IHC in PMID: 12480978; Our standard laboratory procedure included the use of no primary antibody reagent control.<br>Rabbit anti-mouse CD163; Santa Cruz Biotech #sc-33560; Antibody validated for immunocytochemistry by manufacturer; Our standard laboratory procedure included the use of no primary antibody reagent control.<br>Rat anti-mouse CD206; AbD Serotec #MCA2235; Antibody validated for immunohistochemistry by manufacturer using mouse lymph nodes as a positive control; Our standard laboratory procedure included the use of no primary antibody reagent control.<br>Rat anti-mouse CD4; Biolegend #100401; Antibody validated by manufacturer using flow cytometry; Our standard laboratory procedure included the use of no primary antibody reagent control.<br>Rat anti-mouse Ly6B.2; Biorad #MCD771G; Antibody source reference PMID: 6618532; Our standard laboratory procedure included the use of no primary antibody reagent control.<br>Rabbit anti-human Collagen type 1; Chemicon #AB745; Antibody validated for immunohistochemistry by manufacturer and PMID: 1705376; Our standard laboratory procedure included the use of no primary antibody reagent control.<br>Goat anti- Collagen type 3; Southern Biotech #1330; Antibody validated for cross-adsorption using collagen type 1, 2, 4-6 and ELISA by manufacturer and for IHC by PMID: 8943737; Our standard laboratory procedure included the use of no primary antibody reagent control.<br>Goat anti- Collagen type 5; Southern Biotech #1350; Antibody validated for cross-adsorption using collagen type 1-4, 6 and ELISA by manufacturer and for IHC by PMID: 3075544; Our standard laboratory procedure included the use of no primary antibody reagent control.<br>Goat anti- mouse Leukemia Inhibitory Factor; R&D Systems #AB-449; Manufacturer validated antibody for specificity using ELISA, western blot and IHC; We further validated specificity of the antibody using a) no primary antibody reagent control b) concentration matched normal goat IgG control (R&D #AB-108-C) and c) pre-absorption with recombinant mouse LIF.<br>Mouse anti- human developmental myosin heavy chain; Novocastra #106304; Validated by manufacturer for IHC in human muscle during fetal period and for mouse by PMID: 2723681; Our standard laboratory procedure included the use of no primary antibody reagent control.<br>Goat anti- mouse CCL2; R&D Systems #AB-479-NA; Validated by manufacturer for western blot and neutralizing assay; We further validated specificity of the antibody using a) no primary antibody reagent control b) concentration matched normal goat IgG control (R&D #AB-108-C) and c) pre-absorption with recombinant mouse CCL2.<br>Rabbit anti-mouse TGF beta; Abcam #ab92486; Validated by manufacturer for western blot and neutralizing assay; Our standard laboratory procedure included the use of no primary antibody reagent control.<br>Rabbit anti- mouse CCR2; Abcam #ab32144; Validated by manufacturer for immunofluorescence and for mouse by PMID: 21730304; Our standard laboratory procedure included the use of no primary antibody reagent control.<br>Mouse anti- mouse Pax7; Developmental Studies Hybridoma Bank product PAX7; Validated by PMID: 9376315 and in house by western blot; Our standard laboratory procedure included the use of no primary antibody reagent control.<br>Rabbit anti- mouse HSP47; Abcam #ab77609; Validated by manufacturer for western blot and by PMID: 24804164; Our standard laboratory procedure included the use of no primary antibody reagent control.<br>Rat anti- mouse Ertr7; Santa Cruz #sc-73355; Validated by manufacturer for western blot using transformed fibroblast cell line; Our standard laboratory procedure included the use of no primary antibody reagent control.<br>Rat anti- mouse CD31; eBioscience #11-0311; Validated by manufacturer for IHC and flow cytometry; Our standard FACS procedure included the use of single and fluorescence minus one controls.<br>Rat anti- mouse CD45; eBioscience #11-0451; Validated by manufacturer for IHC and flow cytometry; Our standard FACS procedure included the use of single and fluorescence minus one controls.<br>Rat anti- mouse CD11b; eBioscience #11-0112; Validated by manufacturer for IHC and flow cytometry; Our standard FACS procedure included the use of single and fluorescence minus one controls.<br>Goat anti- mouse PDGFR alpha; R&D Systems #AF1062; Validated by manufacturer for IHC and neutralization; Our standard laboratory procedure included the use of no primary antibody reagent control.<br>Rat anti- mouse CD16/32; eBioscience #14-0161; Validated by manufacturer for flow cytometry.<br>Rat anti- mouse PDGFR alpha; eBioscience #25-1401; Validated by manufacturer for flow cytometry; Our standard FACS procedure included the use of single and fluorescence minus one controls.<br>Rat anti- mouse Sca-1; eBioscience #15-5981; Validated by manufacturer for flow cytometry; Our standard FACS procedure included the use of single and fluorescence minus one controls.<br>Mouse anti- mouse integrin alpha 7; Medical and Biological Laboratories #K0046-5; Validated by manufacturer for flow cytometry; Our standard FACS procedure included the use of single and fluorescence minus one controls.

Eukaryotic cell lines

Policy information about cell lines

Cell line source(s)	C2C12 mouse muscle myoblast cell line from American Type Culture Collection
Authentication	C2C12 cells were validated by expression of integrin alpha 7 using flow cytometry and testing the ability of cells to fuse in low serum conditions.
Mycoplasma contamination	Cells were not tested for mycoplasma infection

Commonly misidentified lines (See ICLAC register)	N/A

Animals and other organisms

Policy information about studies involving animals; ARRIVE guidelines recommended for reporting animal research

Laboratory animals	Mus musculus, C57BL/10ScSn-Dmdmdx/J and C57BL/10ScSn
Wild animals	N/A
Field-collected samples	N/A
Ethics oversight	All experimentation complied with all relevant ethical regulations for animal testing and research, and the study protocol was approved by the Chancellor's Animal Research Committee at the University of California, Los Angeles.

Note that full information on the approval of the study protocol must also be provided in the manuscript.
